# Innate immunity and hypertension: unveiling inflammatory pathways for novel therapies

**DOI:** 10.3389/fphar.2026.1931176

**Published:** 2026-09-29

**Authors:** Omar Tluli, Alaa Abdelhamid, Yaman Al-Haneedi, Dima Nasrallah, Ali H. Eid

**Affiliations:** 1 College of Medicine, QU Health, Qatar University, Doha, Qatar; 2 Department of Basic Medical Sciences, College of Medicine, QU Health, Qatar University, Doha, Qatar

**Keywords:** adoptive cell therapy, immunity, inflammation, macrophage polarization, pattern recognition receptors, targeted therapy

## Abstract

Hypertension is a leading cause of global cardiovascular morbidity and mortality, yet its primary etiology remains poorly defined. Accumulating evidence increasingly positions inflammation as a key determinant of disease progression, with both innate and adaptive immune mechanisms playing central roles. Under physiological conditions, the immune system safeguards vascular homeostasis through the coordinated activity of immune cells and soluble mediators. Cells of the innate immune system, such as natural killer cells, dendritic cells, macrophages, and neutrophils, detect pathogen- and damage-associated molecular patterns via pattern recognition receptors, while complement proteins and antimicrobial peptides serve as additional effectors of host defense. Perturbations in these pathways, however, precipitate chronic inflammation that contributes to hypertensive pathology. Engagement of toll-like and nucleotide-binding oligomerization domain-like receptors initiates pro-inflammatory cascades that intensify oxidative stress, impair vasodilation, and compromise endothelial function. These alterations are further propagated by activated immune cells and by complement-mediated recruitment of leukocytes, which sustain a deleterious inflammatory milieu. Collectively, these mechanisms contribute to vascular injury and perpetuate elevated blood pressure. Emerging therapies designed to target these immune-inflammatory pathways offer promising opportunities to alleviate vascular dysfunction and ultimately transform the management of hypertension by addressing its immunological underpinnings.

## 1 Introduction

Cardiovascular diseases (CVDs) remain a formidable challenge to global health, exerting a pervasive impact on morbidity and mortality. It accounts for approximately 17.9 million deaths annually, solidifying its position as the leading cause of mortality (WHO, 2023). CVD encompasses a spectrum of conditions, including coronary artery disease, heart failure, peripheral artery disease, stroke, and hypertension. Among these, the latter stands out as one of the most prevalent and significant contributors to CVD deaths worldwide (WHO, 2023).

Hypertension is a prevailing chronic condition characterised by a sustained elevation in arterial blood pressure, defined as measurements equal to or greater than 140/90 mmHg (Madhur et al., 2021). Its prevalence has reached alarming proportions. Recent data indicates that approximately 1.4 billion adults aged 30–79 years had hypertension in 2024, highlighting the immense scale of the problem and the urgency of addressing this public health crisis (WHO, 2025).

Compounding the gravity of the situation is hypertension’s insidious nature. Often referred to as the “silent killer”, it can remain asymptomatic for years, evading detection and leaving individuals vulnerable to severe complications. Alarmingly, nearly half of affected individuals are unaware of their condition, only around 42% receive a diagnosis and treatment, and approximately one in five achieve adequate blood pressure control (WHO, 2025). This lack of awareness underscores the critical need for improved screening, education, and intervention strategies to mitigate its impact on global health.

Hypertension is broadly classified into two types depending on the root cause of the elevated blood pressure. Primary, or essential, hypertension accounts for 90%–95% of cases and lacks a discernible cause, though it is believed to result from complex interactions between genetic predisposition and environmental factors (Oparil et al., 2018). Conversely, secondary hypertension accounts for the remaining of cases, and is caused by underlying medical conditions such as renal or endocrine disorders, obstructive sleep apnea, or vascular abnormalities (Oparil et al., 2018).

The genesis of hypertension is woven with a spectrum of contributors. These include lifestyle factors, such as diet, physical activity and stress, alongside aging and genetic susceptibility. Increasingly, inflammation has been implicated as a critical factor in both the onset and progression of hypertension (Aboukhater et al., 2023). Inflammation represents a biological response to harmful stimuli like pathogens, irritants, or damaged cells. This response is orchestrated by the immune system, a highly intricate system that, when functioning optimally, identifies and eliminates threats while maintaining homeostasis (Yatim and Lakkis, 2015).

The immune system comprises two principal arms, innate and adaptive immunity. These systems act synergistically through sophisticated signaling networks mediated by cytokines—small molecules that coordinate cellular responses (Parkin and Cohen, 2001). Indeed, cytokines are crucial in regulating the intensity and duration of immune responses by promoting cell activation, differentiation, and migration, thereby orchestrating the body’s defense mechanisms against pathogens. However, dysregulation within these systems can transform protective mechanisms into pathological processes. Such immune dysfunction has been strongly associated with vascular inflammation, oxidative stress, and endothelial damage (Dixon et al., 2020). Accordingly, this review explores and critically appraises the accumulating evidence that innate immune pathways contribute to the development and progression of hypertension. It emphasizes how targeting these mechanisms may offer innovative therapeutic strategies for managing this pervasive condition.

## 2 Role of the innate immune system

The innate immune system serves as the body’s first line of defense, providing rapid and nonspecific responses against invading pathogens. It includes physical barriers, like the skin and mucous membranes, which act as formidable shields against pathogen entry (Chaplin, 2003). Beyond these barriers, it employs a diverse array of effector immune cells, including natural killer (NK) cells, dendritic cells (DCs), monocytes/macrophages and neutrophils (Marshall et al., 2018). These phagocytes engulf and eliminate pathogens, while DCs are essential for antigen presentation, activating adaptive immune responses. Simultaneously, NK cells contribute to the direct destruction of infected or abnormal cells.

The efficacy of these processes hinges on the recognition of invading pathogens through pattern recognition receptors (PRRs). These germline-encoded receptors detect conserved molecular signatures of pathogens (pathogen-associated molecular patterns [PAMPs]) and signals from damaged host cells (damage-associated molecular patterns [DAMPs]) (Takeuchi and Akira, 2010). Among PRRs, toll-like receptors (TLRs) and nucleotide-binding oligomerization domain-like receptors (NLRs) are extensively studied for their role in initiating immune responses. Their activation triggers signaling cascades that lead to cytokine release, phagocytosis, and inflammation.

Complement proteins and antimicrobial peptides further augment innate immunity by enhancing pathogen clearance and directly disrupting microbial membranes (Zimmer et al., 2014). Interestingly, recent studies increasingly highlight the involvement of innate immune components in hypertension pathogenesis (Guzik et al., 2024; Manzano et al., 2025; Hunter and Harrison, 2026). Indeed, dysregulation of PRRs and inflammatory mediators has been shown to influence vascular function and blood pressure regulation. A key development in this field is the recognition that hypertensive stimuli, such as mechanical stretch and high dietary sodium, can prime innate immune cells and insitgate inflammation. This process involves the activation of intracellular signaling pathways, leading to the production of pro-inflammatory cytokines and the formation of immunogenic neoantigens, which drive immune responses that ultimately perpetuate hypertension (Shokoples et al., 2024). Therefore, targeting these components offers promising therapeutic interventions for hypertension management.

Despite the growing body of evidence linking innate immune activation to hypertension, the direction and magnitude of this relationship remain incompletely resolved. A central question is whether innate immune activation represents an initiating driver of blood pressure elevation or acts as an amplifier of vascular, renal, and neural injury once hypertension has developed. Experimental studies support a causal contribution, as genetic or pharmacological disruption of selected inflammatory pathways can attenuate blood pressure elevation in several models; however, these findings are highly dependent on the experimental stimulus, tissue examined, and timing of intervention. Human evidence also remains predominantly associative, with increased inflammatory markers and altered innate immune-cell profiles observed in hypertensive individuals but relatively limited evidence demonstrating that these changes precede hypertension or are sufficient to cause it. Thus, innate immunity should not be viewed as a single, uniformly causal pathway; rather, it is more appropriately considered a context-dependent contributor that may participate in initiation, amplification, or maintenance of hypertension depending on the underlying phenotype and hypertensive stimulus. This distinction is particularly important when considering therapeutic translation, because successful intervention may require identification of patients in whom immune activation is a dominant pathogenic mechanism rather than targeting inflammation indiscriminately.

## 3 Role of the innate immune system in hypertension

### 3.1 Toll-like receptors

TLRs play a critical role in the initiation of inflammation, thereby playing a crucial role in the pathogenesis of hypertension. These receptors are transmembrane proteins with an extracellular domain for ligand recognition, a single transmembrane helix, and an intracellular Toll/interleukin-1 receptor (TIR) domain that mediates downstream signaling. This structural organizations underpins their functional versatility.

In humans, 10 TLRs (1–10) have been identified. These are categorized into two main groups based on their cellular localization: cell surface TLRs (TLRs 1,2,4-6, and10) and endosomal intracellular TLRs (TLRs 3,7–10) (Gay et al., 2014; Mourits et al., 2020). The distinction in localization reflects their ligand specificities and signaling mechanisms. Cell surface TLRs predominantly recognize bacterial components such as lipopolysaccharides, while endosomal TLRs detect nucleic acids from pathogens or damaged cells.

Upon activation of TLRs 1 through 9, two primary cascades are engaged: the myeloid differentiation primary response 88 (MyD88) and the TIR-domain-containing adapter inducing interferon-β (TRIF) (Figure 1). MyD88 is a universal adapter protein for all TLRs except TLR3, while TRIF is exclusively engaged by TLR3 and partially by TLR4. These pathways converge on transcription factors such as Nuclear Factor kappa B (NF-κB) and interferon regulatory factors, culminating in the transcriptional activation of genes encoding pro-inflammatory cytokines, chemokines, and co-stimulatory molecules. This orchestrated response amplifies inflammation and recruits immune effector cells. (Kawasaki and Kawai, 2014). In marked contrast to the pro-inflammatory roles of other TLRs, TLR10 exhibits anti-inflammatory properties by virtue of its ability to suppress MyD88 and TRIF pathways (Jiang et al., 2016; Mourits et al., 2020).

**FIGURE 1:**
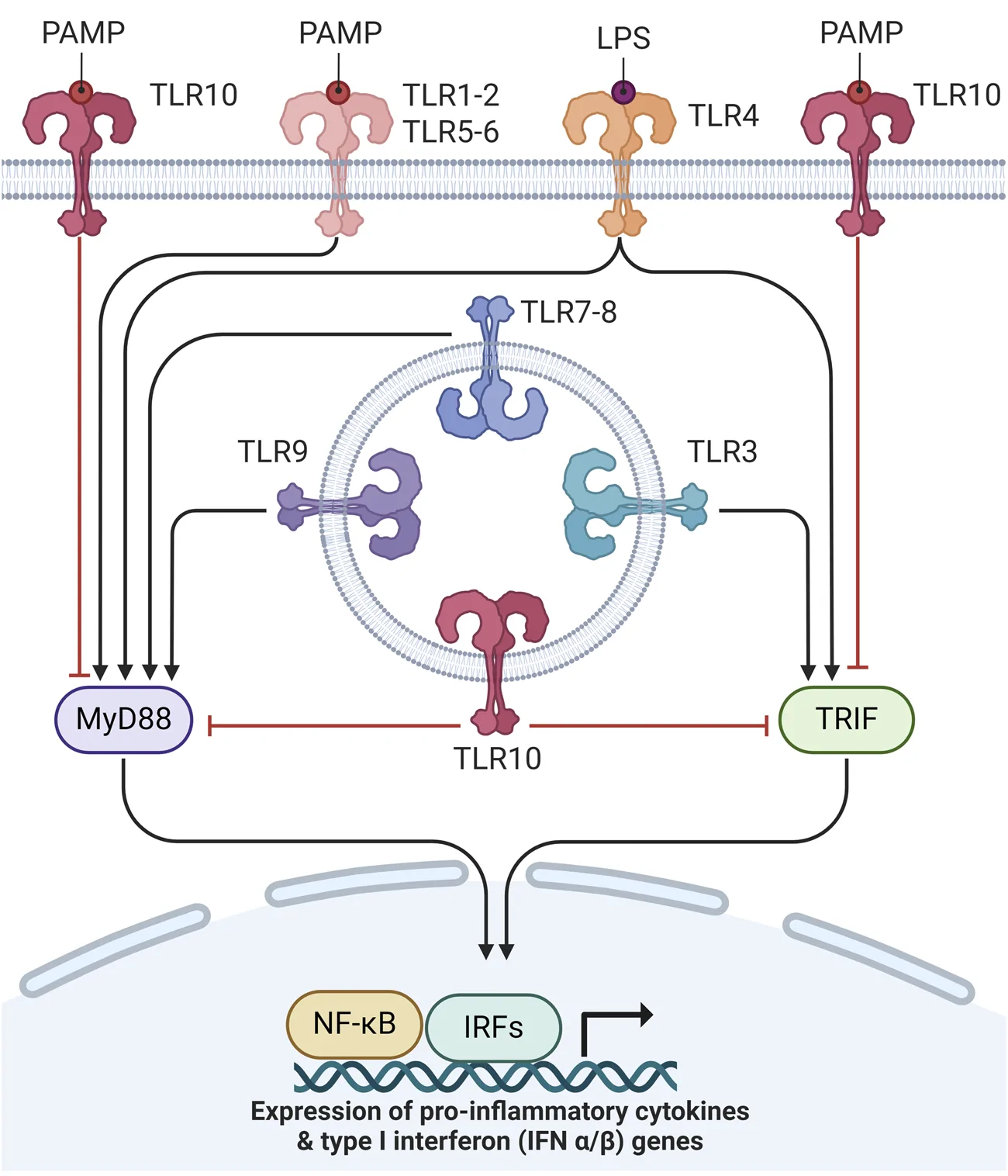
Activation of TLRs by PAMPs, such as LPS, and their downstream signaling pathways in immune response. TLRs, including TLR1-2, TLR4, TLR5-6, TLR7-8, TLR9, and TLR3, are located on the cell surface or within endosomes and recognize distinct PAMPs. Upon ligand binding, TLRs recruit adaptor proteins: MyD88 for most TLRs (except TLR3) and TRIF for TLR3 and TLR4, initiating intracellular signaling cascades. These pathways converge on key transcription factors, NF-κB and IRFs, which translocate to the nucleus and drive the expression of pro-inflammatory cytokines and type I interferons (IFN α/β), thereby orchestrating innate immune responses against microbial pathogens. (Created in BioRender. Abdelhamid et al. (2026) https://BioRender.com/52as2c2). Abbreviations: TLR, Toll-like receptor; PAMP, pathogen-associated molecular pattern; LPS, lipopolysaccharide; MyD88, myeloid differentiation primary response protein 88; TRIF, TIR-domain-containing adaptor-inducing interferon-β; NF-κB, nuclear factor kappa B; IRF, interferon regulatory factor; IFN, interferon.

#### 3.1.1 TLRs in neuronal tissue: their role in hypertension

TLRs are increasingly recognized as key modulators in the pathogenesis of hypertension, influencing both its initiation and progression through sympathetic regulation and inflammatory pathways. The central nervous system, particularly the paraventricular nucleus (PVN) of the hypothalamus, serves as a critical locus for blood pressure regulation via mechanisms such as baroreflex control, body fluid homeostasis and sympathetic outflow (Dampney et al., 2018). Intriguingly, studies in hypertensive rats uncovered an increased expression of TLR4 in the PVN, underscoring their potential involvement in the development of hypertension. Accordingly, blocking TLR4 has been shown to decrease sympathetic activity and therefore lower blood pressure, highlighting its therapeutic potential (Masson et al., 2015). Additionally, the evident upregulation and activation of TLR 7,8 and 9 pathways in the PVN of pre-hypertensive rats may implicate these receptors in priming pro-inflammatory responses prior to the onset of hypertension (Lazaridis et al., 2021).

#### 3.1.2 TLRs in vascular smooth muscle cells: their role in hypertension

TLRs are constitutively expressed on endothelial and vascular smooth muscle cells (VSMCs), prompting speculation about their involvement in vascular dysfunction and hypertension. Substantiating this, TLR4 is notably upregulated in models of Angiotensin II (Ang II)-induced hypertension (Nakashima et al., 2015). Interestingly, activation of TLR4 suppresses antioxidant enzymes while stimulating NADPH oxidase activity, leading to increased reactive oxygen species (ROS) production. This cascade amplifies pro-inflammatory cytokine expression and exacerbates vascular injury through oxidative stress and endothelial dysfunction. These processes collectively contribute to vascular remodeling and hypertensive pathology (Nakashima et al., 2015). In contrast, pharmacological inhibitors of TLR4 elicit opposing and rather ameliorative effects, supporting the role of TLR4 in altering vascular properties and contributing to hypertension (Dange et al., 2015). Similarly, both TLR2 and TLR9 have also been associated with vascular dysfunction through pro-inflammatory and pro-oxidative signaling pathways. Indeed, these receptors amplify ROS production while also reducing endothelial nitric oxide (NO) bioavailability, a potent vasodilator, further aggravating vascular dysfunction and hypertension (Roshan et al., 2016).

#### 3.1.3 TLRs in the renal system: their role in hypertension

The renal system is another critical site where TLRs influence hypertensive mechanisms. Ang II-induced activation of TLR4 within renal epithelial, mesangial, and tubular cells drives NF-κB signaling and enhances the release of pro-inflammatory cytokines such as tumor necrosis factor-alpha (TNF-α) and interleukin-6 (IL-6) (Kang et al., 2009; Xu et al., 2017). These inflammatory mediators exacerbate renal damage and contribute to elevated blood pressure. Studies involving TLR4 knockout mice have shown a diminished hypertensive response to Ang II (Pushpakumar et al., 2017). Conversely, mice with intact TLR4 exhibit heightened inflammatory activity and increased blood pressure. Notably, pharmacological antagonism of TLR4 effectively mitigates these effects, demonstrating its pivotal role in renal inflammation and hypertension (Zhang et al., 2015; Pushpakumar et al., 2017).

### 3.2 Nucleotide-binding oligomerization domain

NLRs constitute another class of intracellular PRRs implicated in hypertension. These molecules are crucial contributors to host defense and cellular stress responses, and are expressed in a variety of cell types, including immune cells, epithelial cells, fibroblasts, and endothelial cells (Wicherska-Pawłowska et al., 2021). These receptors are characterized by their NACHT domain for oligomerization upon activation, an LRR domain for ligand recognition, and an N-terminal effector domain that mediates downstream signaling (Platnich and Muruve, 2019). NLRs are broadly categorized into canonical inflammasome-forming members (e.g., NLRP3, NLRC4) and non-canonical members that function independently or through alternative pathways (Figure 2). Canonical NLRs such as NLRP3 assemble inflammasomes upon activation by PAMPs, DAMPs, oxidative stress, or mitochondrial dysfunction. Inflammasomes subsequently activate caspase-1 to cleave the pro-inflammatory cytokines interleukin-1 beta (IL-1β) and interleukin-18 (IL-18) into their active forms, amplifying inflammatory responses (Broz and Monack, 2013; Bulté et al., 2023). However, the synthesis of these pro-inflammatory cytokines requires a priming step, through TLR-mediated activation of NF-κB. Conversely, the non-canonical pathway involves NLRs such as NOD1, NOD2, NLRP6, NLRP12, and NLRP3, functioning through inflammasome-dependent and independent mechanisms (Platnich and Muruve, 2019). These receptors respond to cytosolic lipopolysaccharide from Gram-negative bacteria, leading to the activation of caspase-4/5/11 and the induction of pyroptosis, a unique form of inflammatory cell death (Broz and Dixit, 2016). Moreover, the non-canonical pathway contributes to host defense by promoting the activation of inflammatory mediators such as NF-κB, akin to that observed with TLRs (Wang et al., 2015).

**FIGURE 2:**
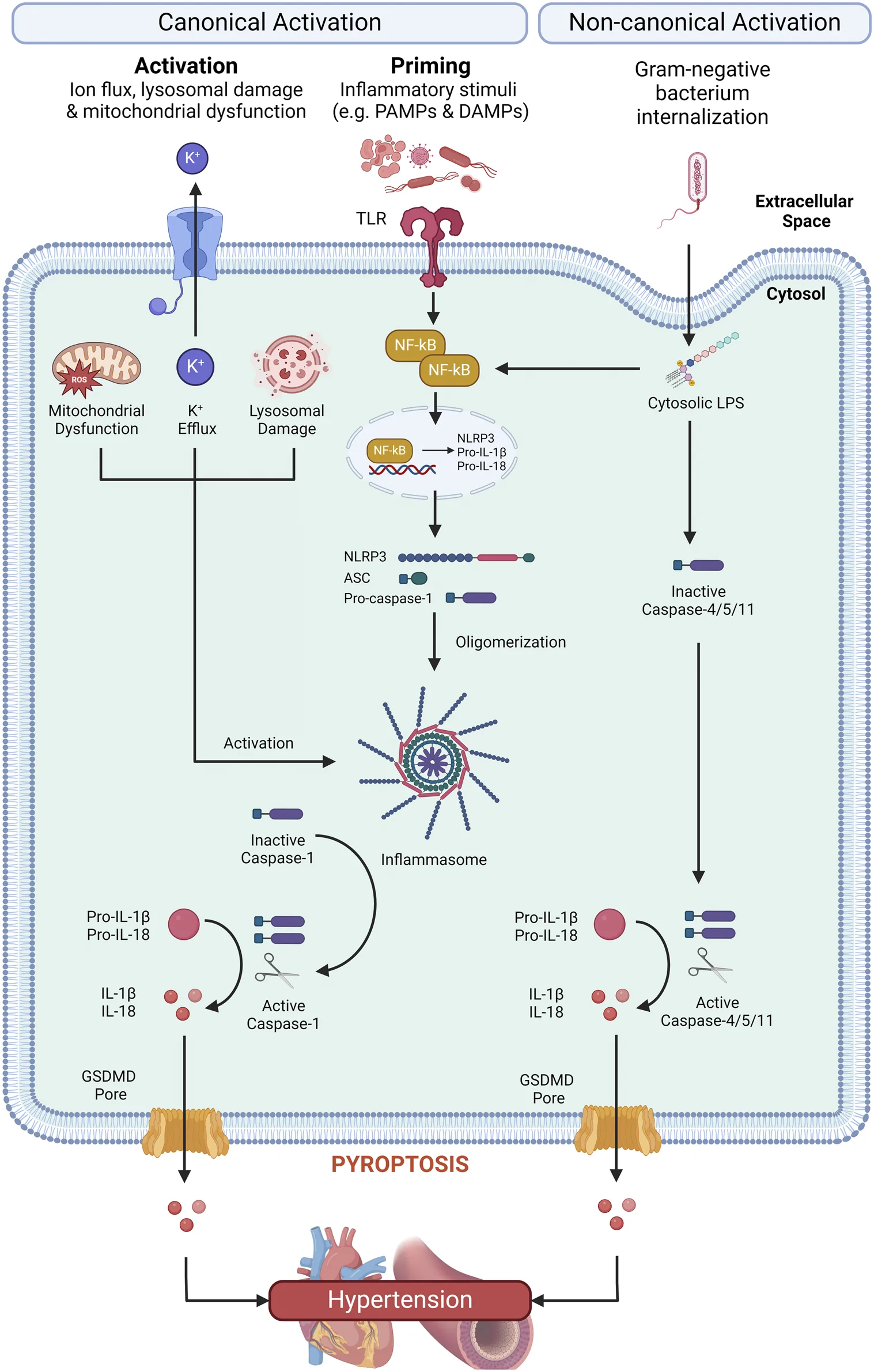
Canonical and Non-canonical pathways of inflammasome activation, leading to pyroptosis and inflammation, with implications for hypertension. In the canonical pathway, cellular stress signals such as ion flux (e.g., K^+^ efflux), lysosomal damage, or mitochondrial dysfunction activate the NLRP3 inflammasome, which is primed by inflammatory stimuli like PAMPs and DAMPs via TLR signaling and NF-κB activation. This leads to the assembly of the inflammasome complex, comprising NLRP3, ASC, and pro-caspase-1, resulting in caspase-1—resulting in caspase-1 activation. Active caspase-1 cleaves pro-IL-1β and pro-IL-18 into their mature, bioactive forms and triggers GSDMD pore formation, inducing pyroptosis, a pro-inflammatory form of cell death. In the non-canonical pathway, cytosolic LPS from internalized Gram-negative bacteria directly activates caspase-4/5/11, which similarly cleaves GSDMD and pro-IL-1β, leading to pyroptosis and cytokine release. Both pathways culminate in the release of inflammatory mediators and pyroptotic cell death, contributing to systemic inflammation and pathological conditions such as hypertension. (Created in BioRender. Abdelhamid et al. (2026) https://BioRender.com/1h3va88). Abbreviations: NLRP3, NOD-, LRR-, and pyrin domain-containing protein 3; ASC, apoptosis-associated speck-like protein containing a caspase recruitment domain; PAMP, pathogen-associated molecular pattern; DAMP, damage-associated molecular pattern; TLR, Toll-like receptor; NF-κB, nuclear factor kappa B; IL, interleukin; GSDMD, gasdermin D; LPS, lipopolysaccharide.

#### 3.2.1 NLRs in hypertension

Among the several inflammasomes contributing to hypertension, NLRP3 stands out for its prominent pro-inflammatory effects. Activation of NLRP3 by PAMPs, DAMPs, oxidative stress, potassium efflux, mitochondrial dysfunction, lysosomal damage, or acute phase reactants leads to elevated levels of IL-1*β* and IL-18, cytokines strongly associated with hypertensive pathophysiology (Villegas et al., 2013; Pasqua et al., 2018; Xiao and Harrison, 2020). This aligns with the elevated serum levels of IL-1β and expression of IL-18 receptor accessory protein, a subunit of IL-18 receptor, observed in hypertensive patients (Dalekos et al., 1997; Alexander et al., 2019; Griendling et al., 2021). This inflammatory milieu is driven by upstream signaling pathways that initiate NLRP3 activation in response to hemodynamic stress. For instance, endothelial stretch from elevated blood pressure triggers the release of damage-associated signals such as extracellular ATP (eATP), which activate the P2X7 receptor (P2RX7) on antigen-presenting cells, thereby serving as a potent trigger of NLRP3 activation (Zhao et al., 2019; Shokoples et al., 2021). In line with this mechanism, pharmacological inhibition or genetic deletion of P2RX7 reduces blood pressure and vascular inflammation in hypertensive mice models (Shokoples et al., 2023). In parallel, endothelial stretch activates the growth arrest-specific 6 (GAS6)/Axl pathway, which promotes the differentiation of monocytes into pro-inflammatory DCs that produce IL-1β in an NLRP3-dependent manner (Van Beusecum et al., 2021). Collectively, these pathways link mechanical stress and immune activation to NLRP3 inflammasome signaling in hypertension. The downstream consequences of NLRP3 activation are evident at the vascular level. Murine models of Ang II-induced hypertension have demonstrated increased inflammasome activity alongside vascular remodeling characterized by phenotypic switching of VSMCs from contractile to proliferative states. This transition is accompanied by increased matrix production, cell proliferation and migration, thereby contributing to vascular remodelling, arterial stiffness and sustained elevation of blood pressure (Sun et al., 2017; Elmarasi et al., 2024).

Therapeutic targeting of NLRP3 has shown promise not only in reducing blood pressure but also in alleviating renal inflammation and fibrosis associated with hypertension (Krishnan et al., 2019). Similarly, inhibiting NF-κB provides the dual benefit of lowering blood pressure and protecting against renal injury in hypertensive rats (Zambom et al., 2019). Taken together, these findings highlight the multifaceted contributions of canonical and non-canonical NLR pathways to hypertensive disease progression, warranting further exploration in the development of targeted therapeutic strategies.

### 3.3 Monocytes/macrophages

Monocytes and macrophages, both parts of the mononuclear phagocyte system, are versatile immune cells with distinct yet intertwined functions. Monocytes, originating in the bone marrow, are released into the bloodstream where they circulate until recruited to sites of inflammation. This recruitment is facilitated by various adhesion and chemotactic molecules, notably CC chemokine ligand 2, also known as monocyte chemoattractant protein-1, which binds to its receptor CC chemokine receptor 2 (CCR2) (Yerra and Advani, 2022). Once at the site, monocytes differentiate into macrophages, phagocytes adept at engulfing and eliminating pathogens, apoptotic cells, and cellular debris. Macrophages exhibit remarkable functional plasticity in response to local environmental cues and play a central role in modulating immune responses. Although they are commonly classified into broadly defined M1 and M2 subsets, this classification represents a simplified framework, as macrophage activation occurs along a continuum of dynamic functional states *in vivo* (Figure 3).

**FIGURE 3:**
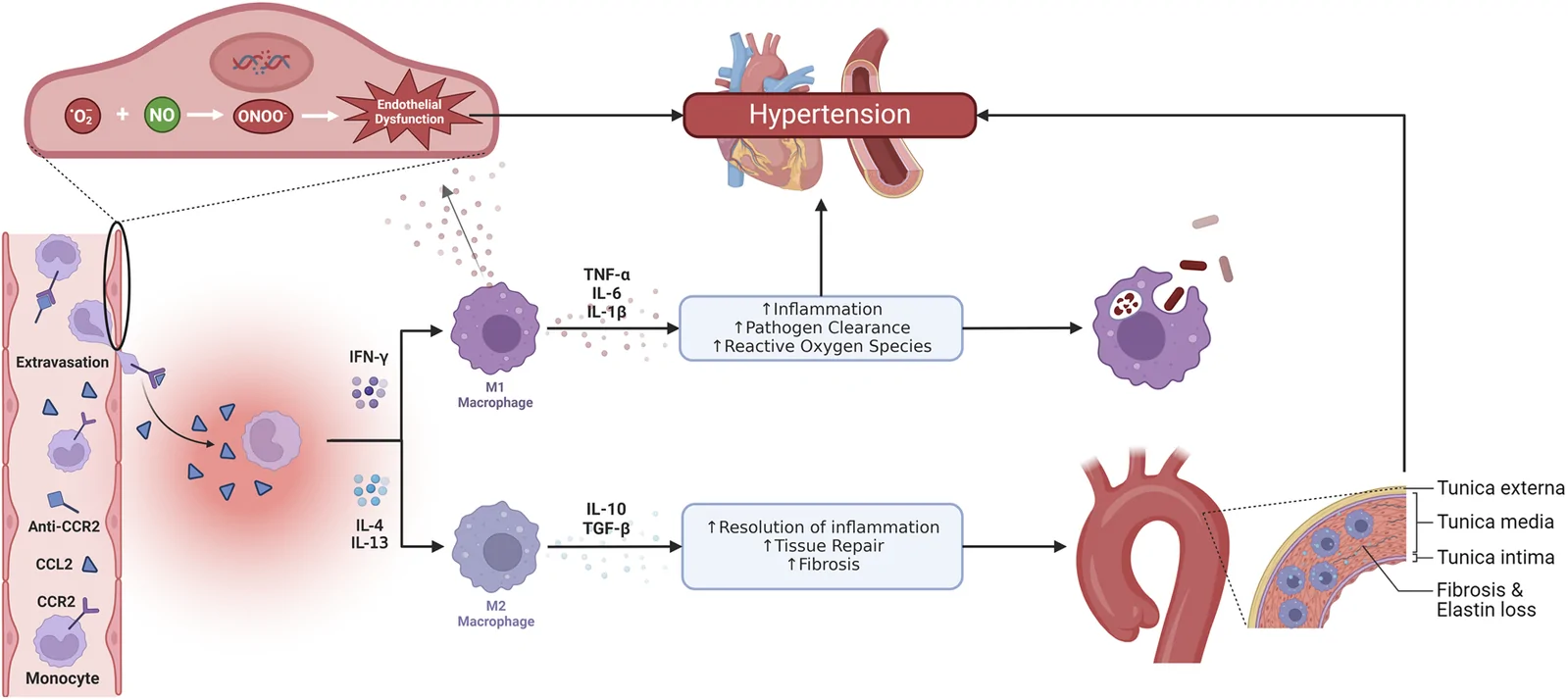
Role of macrophage polarization and inflammatory signaling in the development and progression of hypertension. The figure highlights the interplay between endothelial dysfunction, immune cell activation, and vascular remodeling. Oxidative stress, driven by the reaction between O_2_^−^ and NO to form ONOO^−^, leads to endothelial dysfunction, a key contributor to hypertension. This dysfunction promotes monocyte extravasation into tissues via chemokine signaling, particularly through CCL2 binding to CCR2 on monocytes. Once recruited, monocytes differentiate into macrophages that can polarize into pro-inflammatory M1 or anti-inflammatory M2 phenotypes depending on cytokine signals: IFN-γ drives M1 polarization, producing TNF-α, IL-6, and IL-1β, which amplify inflammation, enhance pathogen clearance, and increase reactive oxygen species, further exacerbating vascular damage and contributing to hypertension. In contrast, IL-4 and IL-13 promote M2 polarization, leading to the release of IL-10 and TGF-β, which support resolution of inflammation, tissue repair, and fibrosis. However, chronic inflammation and imbalance between M1 and M2 responses result in structural changes in blood vessels, including loss of elastin and collagen deposition in the tunica intima, media, and externa, ultimately promoting vascular stiffness and sustained hypertension. (Created in BioRender. Abdelhamid et al. (2026) https://BioRender.com/s9m60c1). Abbreviations: O_2_^−^, superoxide; NO, nitric oxide; ONOO^−^, peroxynitrite; CCL2, CC chemokine ligand 2; CCR2, CC chemokine receptor 2; IFN-γ, interferon gamma; TNF-α, tumor necrosis factor alpha; IL, interleukin; TGF-β, transforming growth factor beta.

Within this framework, M1 macrophages are inflammatory powerhouses activated by various pro-inflammatory cytokines, most notably interferon gamma (IFN-γ). They promote inflammation and defend against pathogens through the expression of pro-inflammatory cytokines such as IL-1β, IL-6, and TNF-α (Nathan et al., 1983). M1 macrophages also excel in phagocytosis, generating ROS to eliminate pathogens and promote inflammation against intracellular threats. Conversely, M2 macrophages adopt an anti-inflammatory profile prompted mainly by interleukin-4 (IL-4) and interleukin-13. These macrophages secrete anti-inflammatory cytokines such as interleukin-10 and transforming growth factor beta (TGF- β) (Stein et al., 1992). Beyond immunoregulation, M2 macrophages contribute to tissue repair, fibrosis, and resolution of inflammation. The balance between M1 and M2 macrophages is integral to immune homeostasis, preventing dysregulation implicated in diseases like hypertension.

Of note, the M1/M2 framework is useful as a conceptual starting point but should not be interpreted as a strict biological classification of macrophages *in vivo*. Macrophages can simultaneously express features traditionally attributed to both M1 and M2 states and can dynamically change their transcriptional and functional programs in response to changes in their local environment (Martinez et al., 2008). Contemporary approaches therefore increasingly describe macrophage activation as a continuum or multidimensional spectrum rather than a binary M1/M2 state, with substantial tissue-specific heterogeneity and plasticity (Murray and Wynn, 2011). This distinction is particularly relevant to hypertension, in which macrophages reside in anatomically and functionally distinct compartments, including the kidney, vascular wall, perivascular adipose tissue, heart, and central nervous system, and may acquire different phenotypes according to the prevailing neurohormonal, metabolic, and inflammatory environment. Accordingly, the M1/M2 terminology used in this review is intended to describe broad functional tendencies rather than discrete macrophage lineages, and future studies should incorporate tissue-specific phenotyping, transcriptomic approaches, and functional characterization to define the macrophage states that are actually associated with hypertension.

#### 3.3.1 Macrophage imbalance in hypertension

An increase in M1 macrophages, coupled with a decrease in M2 macrophages, fosters an inflammatory environment conducive to hypertension. This imbalance results from the adverse impact of M1 macrophages on endothelial cells, disrupting their NO regulation. Specifically, M1 macrophage-derived ROS react with endothelial NO, forming peroxynitrite, a highly reactive and detrimental molecule (Vaziri et al., 1999). This reaction damages endothelial cells and reduces NO bioavailability, thereby contributing to hypertension and other CVDs. Conversely, M2 macrophages accumulate in the aortic walls of Ang II-induced hypertensive mice, contributing to fibrosis and elastin loss, ultimately increasing blood pressure (Moore et al., 2015). Accordingly, inhibiting CCR2, essential for macrophage recruitment and activation, in these models reduces M2 macrophage aortic accumulation. This intervention resulted in a decrease in collagen deposition, mitigation of elastin loss, and a resolution of hypertension (Moore et al., 2015).

The imbalance between M1 and M2 macrophages in hypertension is linked to neurohormonal signaling and metabolic reprogramming. The renin–angiotensin system (RAS), a central regulator of blood pressure, directly influences macrophage polarization. Angiotensin II has been shown to induce M1 polarization through activation of the angiotensin type 1 receptor (AT1R) and subsequent downstream HIF-1α and NF-κB signaling (Yang et al., 2022). This establishes a mechanistic link between RAS and pro-inflammatory immune activation. In addition, metabolic reprogramming in hypertension further contributes to macrophage polarization. Under physiological conditions, M2 macrophages primarily depend on the tricarboxylic acid cycle and oxidative phosphorylation, whereas M1 macrophages rely predominantly on glycolysis for energy production (Wang et al., 2022). Factors in hypertension, such as HIF-1α stabilization seen in hypoxia, increased ROS, IL-1β, and lipid synthesis, induce a metabolic shift from the tricarboxylic acid cycle and oxidative phosphorylation to glycolysis, thereby promoting a skew towards M1 macrophage polarization (Mouton et al., 2020). Hence, targeting these elements could create an anti-inflammatory milieu conducive to the treatment of hypertension.

### 3.4 Dendritic cells

DCs are pivotal players in the immune system, acting as sentinels that bridge innate and adaptive immune responses. Their primary function is to capture, process, and present antigens to T cells, B cells, and NK cells (McCarthy et al., 2021). DCs are stimulated by PAMPs and DAMPs through their PRRs, where they then undergo a process of maturation from immature antigen-capturing cells into mature antigen-presenting cells (APCs). This involves the upregulation of their co-stimulatory B7 molecules, migration to lymphoid organs, and secretion of cytokines, critical steps for their professional antigen-presenting activities (Cella et al., 1997).

#### 3.4.1 Dendritic cells in hypertension

DCs play an important role in salt-sensitive hypertension by virtue of their ability to link sodium-related oxidative stress and innate immune activation to pro-inflammatory immune responses. Indeed, high dietary sodium and hypertensive stimuli promote DC activation and the formation/accumulation of neoatnigens which can then promote antigen presentation and T-cell activation. Activated DCs enhance CD4^+^ T-cell proliferation and promote the production of interleukin-17A, TNF- α, and IFN-γ (Figure 4). These cytokines can further aggravate renal sodium retention, vascular dysfunction, and tissue inflammation, thereby contributing to the development of salt-sensitive hypertension. Along these lines, DCs exhibit increased B7 expression and heightened interaction with CD28 co-stimulatory molecules on pro-inflammatory CD4^+^ T cell subsets. Normally, this activation is counteracted by CTLA4 on CD4^+^ T cells, which exhibits a stronger affinity for B7 on APCs. Accordingly, CTLA4-immunoglobulin administration to Ang II- and DOCA-salt-induced hypertensive mice resulted in a reduction in blood pressure (Vinh et al., 2010).

**FIGURE 4:**
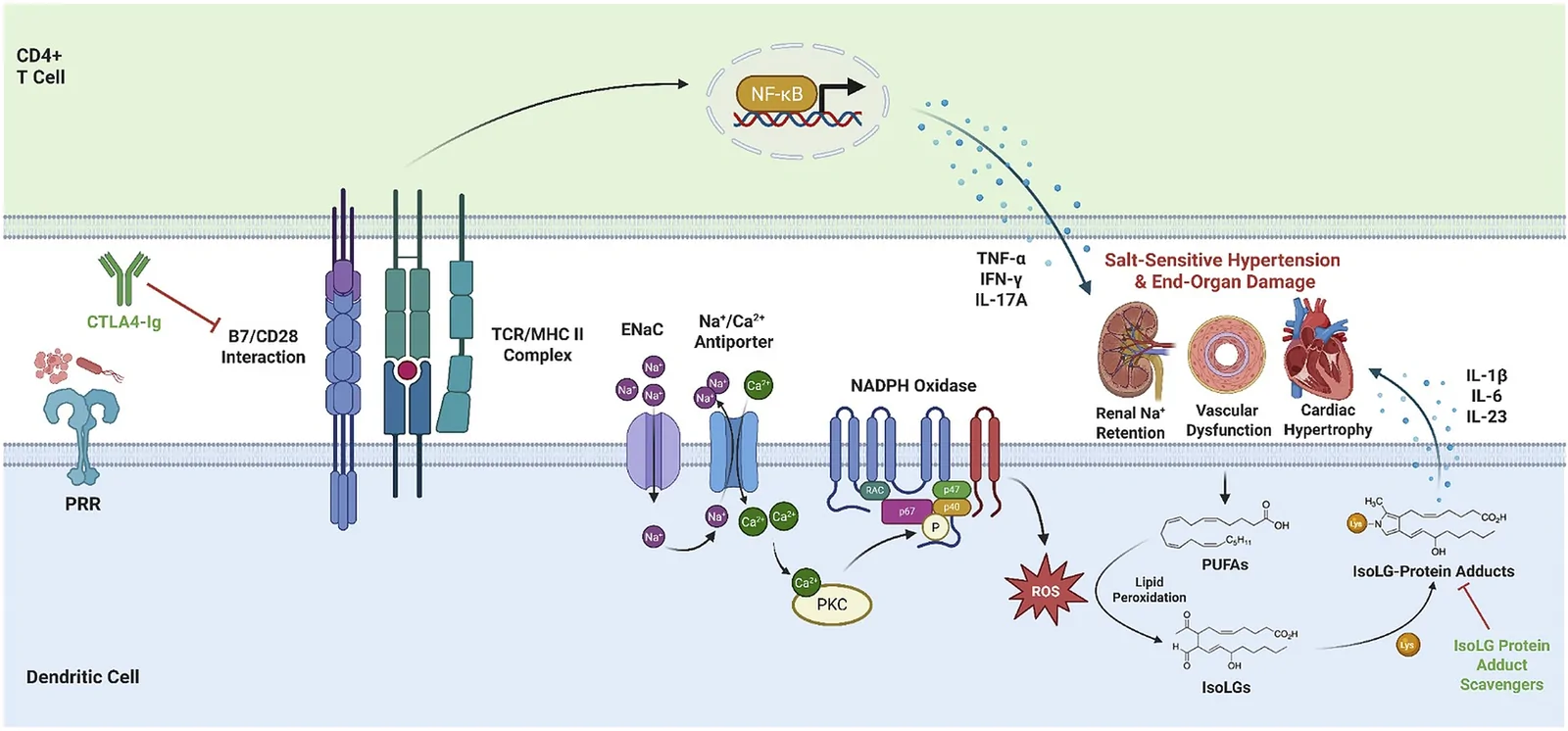
Interaction between DCs and CD4^+^ T cells results in salt-sensitive hypertension and end-organ damage through immune-mediated mechanisms. DCs, upon activation via PRRs, present antigens to CD4^+^ T cells via the TCR/MHC II complex, while costimulatory signals such as B7/CD28 interaction promote CD4^+^ T cell activation. The immunosuppressive agent CTLA4-Ig can block this interaction, modulating immune responses. Activated T cells produce pro-inflammatory cytokines including TNF-α, IFN-γ, and IL-17A, which activate NF-κB signaling in target tissues, promoting inflammation. These cytokines also stimulate ENaC and Na^+^/Ca^2+^ antiporters, leading to increased sodium reabsorption and cellular calcium influx, contributing to renal sodium retention and vascular dysfunction. Concurrently, NADPH oxidase is activated, generating ROS, which induce lipid peroxidation of PUFAs, forming isoLGs. These isoLGs form protein adducts that further exacerbate oxidative stress and inflammation. The resulting cascade promotes renal sodium retention, vascular dysfunction, cardiac hypertrophy, and fibrosis, hallmarks of salt-sensitive hypertension and end-organ damage. Notably, isoLG-protein adduct scavengers may mitigate these effects by neutralizing toxic adducts, highlighting a potential therapeutic strategy. (Created in BioRender. Abdelhamid et al. (2026) https://BioRender.com/kihymoj). Abbreviations: DC, dendritic cell; PRR, pattern recognition receptor; TCR, T-cell receptor; MHC II, major histocompatibility complex class II; CTLA4-Ig, cytotoxic T-lymphocyte-associated protein 4 immunoglobulin; TNF-α, tumor necrosis factor alpha; IFN-γ, interferon gamma; IL, interleukin; NF-κB, nuclear factor kappa B; ENaC, epithelial sodium channel; NADPH, nicotinamide adenine dinucleotide phosphate; ROS, reactive oxygen species; PUFA, polyunsaturated fatty acid; isoLG, isolevuglandin.

Building on this general DC-T-cell mechanisms, recent advancements have identified specific DC subsets with distinct roles in hypertension. For instance, Axl + Siglec-6+ dendritic cells (ASDCs) are a pro-inflammatory population that is markedly increased in the circulation of hypertensive individuals (Villani et al., 2017; Van Beusecum et al., 2021). These cells arise when monocytes are exposed to endothelial cells subjected to hypertensive mechanical stretch. This process is mediated by the release of GAS6 from the endothelium, which activates the Axl receptor on monocytes and drives their differentiation into ASDCs (Van Beusecum et al., 2021). Functionally, ASDCs produce high levels of pro-inflammatory cytokines such as IL-1β, IL-6, and TNF-α and are rich in IsoLG-adducts, enabling them to potently activate T cells (Van Beusecum et al., 2021). Hence, ASDCs serve as a critical link between endothelial mechanical stress and downstream immune activation in hypertension. More importantly, this mechanistic pathway carries therapeutic implications. Targeting the GAS6/Axl axis has been shown to attenuate hypertension and end-organ damage in preclinical models, highlighting the GAS6/Axl axis as a potential therapeutic target in hypertension (Chen et al., 2022).

A major advancement in the understanding of hypertension immunology has been the identification of isolevuglandin (IsoLG)-adducted proteins as key neoantigens that drive DC–mediated T cell activation (Xiao et al., 2018). In the setting of hypertension, increased oxidative stress, particularly in DCs and endothelial cells, leads to the formation of these highly reactive lipid peroxidation products (Davies et al., 2020). IsoLGs covalently modify self-proteins, creating immunogenic neoepitopes that the immune system recognizes as non-self. These IsoLG-adducted proteins are then processed and presented via MHC-I on DCs, providing the necessary antigenic signal for T cell activation, thereby bridging innate and adaptive immunity (Dixon et al., 2017).

The formation of IsoLG-adducts is driven by well-defined intracellular pathways. One mechanism involves an increase in Na^+^ influx into DCs through amiloride-sensitive transporters. Subsequently, Na^+^ is exchanged with Ca^2+^ through Na^+^/Ca^2+^ antiporters, and the heightened intracellular Ca^2+^ levels activate protein kinase C, initiating the phosphorylation and activation of NADPH oxidase, leading to ROS production (Barbaro et al., 2017). These ROS interact with polyunsaturated fatty acids, such as arachidonic acid, culminating in the formation of lipid hydroperoxides, ultimately giving rise to IsoLG. These intermediates covalently bond with lysine residues during post-transcriptional modifications of proteins, eventually giving rise to IsoLG-protein adducts (Xiao et al., 2018). The accumulation of IsoLG-protein adducts in DCs stimulates the release of pro-inflammatory cytokines IL-1β, IL-6, and interleukin-23 (IL-23), contributing to hypertension (Figure 4). Notably, administrating 2-hydroxybenzylamine, scavengers of IsoLG-protein adducts, to Ang II-infused mice prevented vascular inflammation, ameliorated aortic stiffness, and normalized blood pressure, substantiating their significant contribution to vascular remodeling and increased blood pressure (Wu et al., 2016).

Interestingly, specific IsoLG-adducted peptides capable of priming hypertensive responses have been identified. Peptides derived from renal proteins such as SGLT2, Cadherin 16, Kelch domain 7A, and Solute Carrier 23 were shown to bind with high affinity to the murine MHC-I molecule H2-Db, and adoptive transfer of DCs loaded with these IsoLG-adducted peptides was sufficient to prime hypertension in naive mice (Bloodworth et al., 2024). Importantly, this antigen presentation was found to be MHC allele-specific, a finding that may have implications for understanding genetic susceptibility to hypertension in humans (Bloodworth et al., 2024).

The processing of these IsoLG-adducted antigens is critically dependent on the immunoproteasome, a specialized inflammation-induced proteolytic system in DCs (de la Visitacion et al., 2024). In the context of inflammation, IFN-γ and TNF-α replace standard proteasome subunits in DCs with inducible counterparts, leading to the formation of the immunoproteasome, which possesses enhanced proteolytic capacity and is optimized to generate peptides for MHC class I presentation to CD8^+^ T cells (Zou et al., 2025). While this mechanism may contribute to CD8^+^ T-cell-mediated control of intracellular infections, it also plays a role in the immunopathogenesis of several diseases, including hypertension (van den Eshof et al., 2021; Zou et al., 2025). Mechanistically, the immunoproteasome enhances DC presentation of IsoLG-adducted antigens via MHC-I, thereby promoting CD8^+^ T cell activation, which plays a key role in the pathogenesis of hypertension. Notably, pharmacological inhibition and genetic deletion of the immunoproteasome subunit LMP7 (β5i) in murine CD11c^+^ DCs significantly blunted the hypertensive response to Ang II and reduced the accumulation of CD8^+^ T cells in the aorta (de la Visitacion et al., 2024). This highlights the immunoproteasome as a promising therapeutic target to disrupt DC processing of IsoLG-adducted antigens and subsequent T cell activation in hypertension.

### 3.5 Neutrophils

Neutrophils, the predominant white blood cells, are distinguished for their role as the immune system’s first responders. They are recruited to sites of injury, infection, or inflammation, responding to chemotactic factors such as interleukin-8, leukotrienes, N-formyl peptides, complement peptides, and ROS (Futosi et al., 2013). Upon recruitment, they elicit a diverse array of effects as they recognize molecular patterns associated with bacteria and fungi. Subsequently, they engage in phagocytosis, antigen presentation, and the release of ROS, as well as preformed granules containing antimicrobial proteins and enzymes. Moreover, neutrophils can form neutrophil extracellular traps (NETs), which are intricate web-like structures composed of DNA, histones, and antimicrobial proteins that trap and kill microbes (Malech et al., 2020). This process, referred to as NETosis, is dependent on the enzyme peptidyl arginine deiminase 4 (PAD4), which is essential for the unfolding and expulsion of DNA (Figure 5). After fulfilling their functions, neutrophils undergo apoptosis. The elimination of apoptotic neutrophils is facilitated by macrophages, which engulf and clear these dying cells through a process called efferocytosis (Greenlee-Wacker, 2016). The disruption in this precise regulation of neutrophil activities has been linked to several diseases, one of which is hypertension.

**FIGURE 5:**
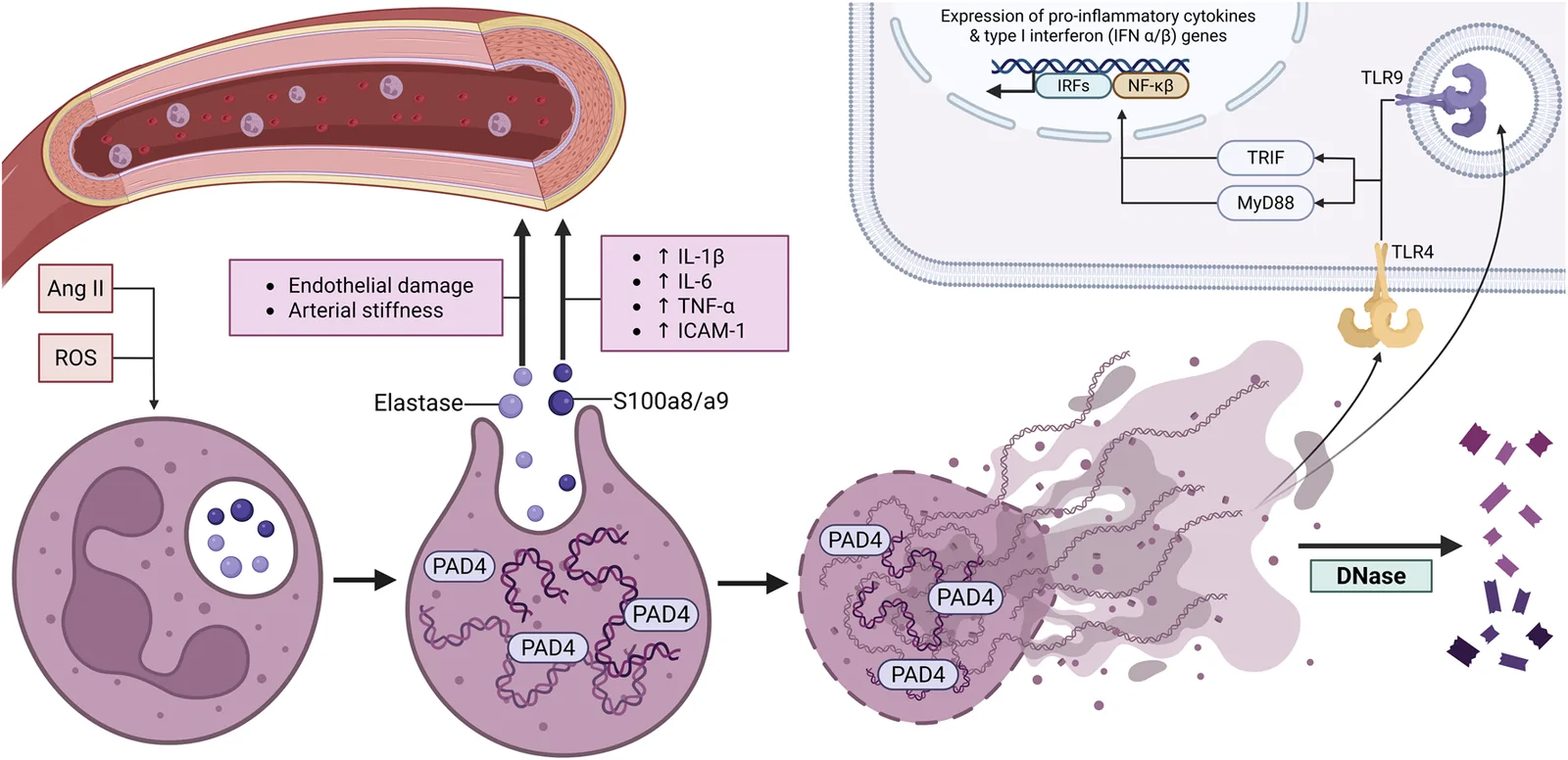
Role of Neutrophil activation and NETosis in promoting vascular inflammation and endothelial dysfunction, contributing to hypertension and arterial stiffness. Ang II and ROS activate neutrophils, triggering the release of elastase and S100A8/A9 proteins, which induce endothelial damage and increase arterial stiffness. Concurrently, these stimuli promote the activation of PAD4, an enzyme that catalyzes histone citrullination, leading to chromatin decondensation and NET formation. The released NETs consist of DNA, histones, and antimicrobial proteins, which can be degraded by DNase. However, excessive or persistent NETs contribute to inflammation by activating TLR4 and TLR9 .via their components, such as DNA and modified histones, leading to downstream signaling through MyD88 and TRIF pathways. This activates NF-κB and IRFs, resulting in the expression of pro-inflammatory cytokines (IL-1β, IL-6, TNF-α) and adhesion molecules (ICAM-1), further amplifying vascular inflammation and endothelial injury. Together, this cascade links NETosis to chronic vascular inflammation, endothelial dysfunction, and the development of hypertensive complications. (Created in BioRender. Abdelhamid et al. (2026) https://BioRender.com/sev5ty1). Abbreviations: Ang II, angiotensin II; ROS, reactive oxygen species; PAD4, peptidylarginine deiminase 4; NET, neutrophil extracellular trap; DNase, deoxyribonuclease; TLR, Toll-like receptor; MyD88, myeloid differentiation primary response protein 88; TRIF, TIR-domain-containing adaptor-inducing interferon-β; NF-κB, nuclear factor kappa B; IRF, interferon regulatory factor; IL, interleukin; TNF-α, tumor necrosis factor alpha; ICAM-1, intercellular adhesion molecule 1.

#### 3.5.1 Neutrophils in hypertension

The clinical relevance of neutrophils in hypertension is supported by human studies showing that elevated neutrophil counts were associated with increased systolic blood pressure, diastolic blood pressure, and pulse pressure (Siedlinski et al., 2020). Additionally, elevated neutrophil-to-lymphocyte ratio significantly correlated with an increased risk of developing hypertension in several populations (Liu et al., 2015; Jhuang et al., 2019). These findings underscore neutrophils not only as predictive biomarkers of hypertensive risk but also as key players and potential therapeutic targets in hypertension.

##### 3.5.1.1 Influence of neutrophils on vascular tone

Under physiological conditions, neutrophils contribute to blood pressure regulation by maintaining vascular tone, primarily through suppressing inducible nitric oxide synthase (iNOS) activity and consequently limiting nitric oxide (NO) production. Accordingly, neutropenia in healthy mice results in increased NO bioavailability and subsequent hypotension, demonstrating the importance of neutrophils in maintaining normal vascular tone (Morton et al., 2008). At first glance, this appears inconsistent with findings that neutrophils isolated from hypertensive rats exhibit increased iNOS activity, which would be expected to enhance NO production and promote vasodilation (Chatterjee et al., 2009). However, this apparent paradox is resolved by the concurrent upregulation of myeloperoxidase (MPO), which offsets the effects of increased iNOS by consuming NO and promoting oxidative stress. MPO, a heme-containing enzyme crucial for ROS production, serves as a biomarker for the infiltration of neutrophils in vascular injury and elevated blood pressure (Baldus et al., 2003; Lau and Baldus, 2006; Brevetti et al., 2008; Fang et al., 2013; Liu et al., 2015). MPO-derived ROS subsequently contributes to oxidative stress, endothelial dysfunction, and vascular inflammation. Moreover, MPO can directly contribute to hypertension by breaking down NO to nitrite, thereby increasing vascular tone (Baldus et al., 2004). In line with this, inhibition of MPO increases NO bioavailability and reduces endothelial dysfunction (Rocha-Penha et al., 2017).

##### 3.5.1.2 Neutrophils in a pro-oxidative milieu

Neutrophils contribute to oxidative stress and hypertension by downregulating the expression of heme oxygenase-1 (HO-1) (Alba et al., 2008). HO-1, an antioxidant enzyme, breaks down the pro-oxidant molecule heme into biliverdin, which is ultimately converted into the antioxidant molecule bilirubin. Consequently, a decrease in the expression of HO-1 fosters a pro-inflammatory environment, exacerbating hypertension. Moreover, within the pro-oxidative environment found in hypertension, neutrophils upregulate their expression of adhesion molecules CD11b/CD18 (Hossain et al., 2012). This augmentation promotes their adhesion and rolling along the endothelium, facilitating their subsequent extravasation and infiltration into the vasculature where they exert their pro-inflammatory effects.

##### 3.5.1.3 Neutrophil elastase in hypertension

An imbalance in the activity of neutrophil elastase, a product of neutrophil degranulation, has also been implicated in the development of hypertension (Figure 5). Neutrophil elastase, a member of the serine protease enzyme family, plays a crucial role in microbial defense and pro-inflammatory processes. It facilitates the breakdown of elastin, collagen, fibronectin, and other extracellular matrix proteins, contributing to tissue remodeling and the clearance of pathogens and damaged tissues (Pham, 2008; Wilgus et al., 2013). Therefore, it is not surprising that their heightened levels are correlated with endothelial damage, arterial stiffness, and ultimately hypertension (Ofosu-Appiah et al., 1997; Yasmin et al., 2005).

##### 3.5.1.4 NETosis in hypertension

Increased release of NETs by neutrophils is associated with hypertension. Their augmented release by neutrophils instigates endothelial damage and precipitates dysfunction. This deleterious effect stems from their intrinsic pro-inflammatory, pro-thrombotic, anti-microbial, and cytotoxic properties (Borissoff et al., 2013). This is underscored by the observation that peripheral NET-forming neutrophils are increased in salt-sensitive hypertensive rats compared to their salt-resistant normotensive counterparts (McCarthy et al., 2021). Ang II and ROS emanating from NADPH oxidase are potent triggers for NETosis in hypertension (Figure 5) (Stoiber et al., 2015; Chrysanthopoulou et al., 2021). Furthermore, NETs robustly activate TLR4 and TLR9, initiating signaling cascades that culminate in the activation of NF-κB (McCarthy et al., 2015; Aldabbous et al., 2016). As previously discussed, activation of NF-κB is pivotal in driving the expression of several pro-inflammatory genes, thus playing a decidedly important role in hypertension.

In the physiologic context, the vigilant clearance of NETs is regulated by deoxyribonucleases (DNases), which proffer protective measures to facilitate the resolution of inflammation and prevent excessive tissue damage (Figure 5). These degradative enzymes fall into two families: DNase I and DNase II. These enzymes exert their effects by cleaving internal phosphodiester bonds within the DNA structures that constitute the very scaffold of NETs (Lauková et al., 2020). Intriguingly, hypertensive rats manifest a decrease in DNase I levels, thereby potentially predisposing them to a persistent accrual of NETs. This, in turn, instigates a self-perpetuating pro-inflammatory milieu (McCarthy et al., 2015). Therefore, strategic administration of DNase I analogues presents itself as a therapeutic approach that may mitigate the pro-inflammatory sequelae of NETs in the setting of hypertension.

Another promising therapeutic strategy for hypertension involves inhibiting the NETosis-dependent enzyme PAD4. Notably, genetic knockout of PAD4 in mice treated with Ang II resulted in attenuated hypertension and reduced vascular inflammation (Krishnan et al., 2024). Moreover, the use of Cl-amidine, an irreversible antagonist of PAD4, has demonstrated efficacy in reducing atherosclerosis, vascular damage, and endothelial dysfunction (Knight et al., 2014). Other PAD4 inhibitors like F-amide, GSK199, and GSK484 have also shown significant disruption of NET formation; however, their impact on blood pressure is yet to be uncovered (Luo et al., 2006; Lewis et al., 2015).

Neutrophils, in their multifaceted contribution to the hypertensive milieu, also mediate cardiac inflammation and fibrosis through their release of S100a8/a9 proteins. These proteins, members of the S100 calcium-binding family, are predominantly found in myeloid cells, including neutrophils (Averill et al., 2012). They serve multiple functions, including altering migration and adhesion, facilitating phagocytosis, and enhancing NADPH oxidase activity. For instance, in response to Ang II administration in rats, there was a significant increase in the expression of S100a8/a9 in circulating neutrophils and in the heart (Wu et al., 2014). Interestingly, cardiac fibroblasts exhibit a pronounced upregulation of TLR4, the receptors for S100a8/a9 proteins. Activation of these receptors induces a pro-inflammatory state in fibroblasts, characterized by the release of the cytokines IL-1β, IL-6, and TNF-α (Wu et al., 2014). Moreover, introducing exogenous S100a8/a9 proteins demonstrated an elevation in the expression of adhesion molecules like ICAM-1, fostering the infiltration of neutrophils to sites of inflammation (Viemann et al., 2005). Correspondingly, the administration of anti-S100A9 antibodies to hypertensive mice not only halted the increase in these cytokines, but also prevented perivascular and interstitial fibrosis, the upregulation of profibrotic genes, and myocardial hypertrophy (Kerkhoff et al., 2005).

### 3.6 Natural killer cells

NK cells are lymphocytes with innate cytotoxic potential. Their defining characteristic is their ability to target cells without prior sensitization and their expression of specific cell surface markers such as CD56 and CD16 (Cooper et al., 2001). NK cells can be classified into two main subsets based on CD56 density: CD56^dim^ and CD56^bright^. CD56^dim^ NK cells are cytotoxic and mediate the direct killing of target cells by releasing granules containing perforin and granzymes (Jacobs et al., 2001). In contrast, CD56^bright^ NK cells are primarily immunoregulatory, and produce cytokines like IFN-γ and TNF-α which modulate immune responses and regulate the activity of other immune cells (Jacobs et al., 2001). A delicate balance between these subsets is crucial for immune homeostasis and effective immune responses, and its disruption can lead to pathological conditions.

#### 3.6.1 Natural killer cells in hypertension

NK cells have emerged as key players in the pathogenesis of hypertension. Their role is largely mediated through interactions with monocytes/macrophages, forming a dynamic interplay that amplifies inflammation. These interactions involve receptor-based NK cell-macrophage interactions such as CD154-CD40, 2B4-CD48, and NKG2D-MIC/RAI-1 (Knorr et al., 2014). Additionally, pro-inflammatory cytokines. released by activated macrophages, including interleukin 12, IL-18, and TNF-α, further stimulate NK cell activation (Michel et al., 2012). Once activated, NK cells produce IFN-γ, which triggers the differentiation of monocytes into macrophages or DCs (Goldszmid et al., 2012). This reciprocal communication contributes to a feedforward amplification loop that recruits inflammatory cells to sites of vascular injury.

Beyond immune cell crosstalk, activated NK cells directly promote vascular inflammation and endothelial dysfunction through the release of IFN-γ, TNF-α, perforin, and granzymes. These mediators increase endothelial expression of adhesion molecules, including vascular cell adhesion molecule-1 (VCAM-1) and ICAM-1, facilitating leukocyte adhesion and infiltration into the vascular wall (Kossmann et al., 2013). The resulting inflammatory response promotes oxidative stress, reduces nitric oxide bioavailability, impairs endothelium-dependent vasodilation, and contributes to vascular remodeling and increased arterial stiffness, all of which are hallmarks of hypertension (Kossmann et al., 2013; Drummond et al., 2019).

Experimental studies have further established a causal role for NK cells in hypertension. IFN-γ is a key mediator of Ang II-induced vascular dysfunction, and mice lacking T-bet, the transcription factor required for IFN-γ production, exhibit reduced NK-cell activation, diminished immune-cell recruitment, and protection from Ang II-induced vascular injury (Lazarevic and Glimcher, 2011; Kossmann et al., 2013). Likewise, depletion of NK cells attenuates vascular dysfunction, whereas adoptive transfer of NK cells restores susceptibility to Ang II-mediated vascular damage, highlighting their direct contribution to hypertensive vascular pathology (Kossmann et al., 2013).

Emerging human studies provide translational support for these experimental findings. Immunophenotyping and single-cell transcriptomic analyses have identified altered circulating NK-cell populations characterized by enhanced activation and inflammatory gene expression in individuals with hypertension (Delaney et al., 2021). Furthermore, increased proportions of circulating NK cells have been associated with higher systolic blood pressure in large multi-ethnic cohorts, supporting a role for NK cells in human vascular inflammation and immune dysregulation (Delaney et al., 2021). Collectively, these findings underscore the multifaceted contribution of NK cells to hypertension through their interactions with macrophages, promotion of endothelial dysfunction, and amplification of vascular inflammation, suggesting that targeting NK-cell-mediated immune pathways may represent a promising therapeutic strategy.

#### 3.6.2 Natural killer cells in preeclampsia

Research into the role of NK cells has mainly focused on their involvement in preeclampsia. Within the maternal-fetal interface, these cells, particularly the CD56^bright^ subset, are abundantly present and play a crucial role in vascular remodeling and regeneration (Trundley and Moffett, 2004). Uterine NK cells achieve this through the secretion of various cytokines and angiogenic factors, including interleukin-8, CXC-chemokine ligand 10, vascular endothelial growth factor (VEGF), and placental growth factor. These factors facilitate spiral artery remodeling to meet the increased demands of the developing fetus (Hanna et al., 2006; Kalkunte et al., 2008).

In normal pregnancies, spiral arteries transform from narrow and highly muscularized vessels to less muscularized ones lacking vasomotor responsiveness, a process crucial for maternal-fetal circulation (Rusidzé et al., 2023). However, in preeclampsia, spiral artery remodeling is impaired, leading to reduced blood flow to the placenta. This pathological state correlates with an altered cytokine secretion profile from NK cells extracted from affected women (Fukui et al., 2011). Murine models with deficient or impaired expansion of uterine NK cells support this association; these models exhibit compromised spiral artery remodeling and impaired placental function (Sliz et al., 2019).

The dysregulation of NK receptor expression also plays a role in the pathogenesis of preeclampsia. Killer cell immunoglobulin-like receptors (KIRs) and NKG2A receptors are particularly implicated in this condition (Hiby et al., 2004; El Costa et al., 2009). These receptors modulate NK function through interactions with trophoblasts, specialized embryonic cells critical for placental development. Trophoblasts express HLA-C and HLA-G ligands that engage with KIRs on NK cells to promote immune tolerance. However, downregulation or functional impairment of these ligands results in heightened NK cell cytotoxicity. This fosters inflammation and endothelial dysfunction at the maternal-fetal interface (Rouas-Freiss et al., 1997; Hiby et al., 2004; Hiby et al., 2010).

Elevated secretion of pro-inflammatory cytokines by NK cells further exacerbates preeclampsia. Increased levels of TNF-α and IFN-γ have been observed in affected individuals (Raghupathy, 2013). IFN-γ disrupts immunological tolerance at the placental level while simultaneously reducing vascular endothelial growth factor secretion. This dual effect impairs angiogenesis and worsens placental insufficiency, thereby contributing to the pathogenesis of preeclampsia (Geldenhuys et al., 2018).

### 3.7 The complement system

The complement system is a critical component of the innate immune response, comprising more than thirty proteins that are either membrane-bound or circulating in blood and interstitial fluids (Sarma and Ward, 2011). Its primary functions include “complementing” the bactericidal activities of antibodies and orchestrating the recruitment of additional immune cells to combat invading microorganisms. (Cedzyński et al., 2019). Activation of this system occurs through three distinct pathways: the classical, lectin, and alternative pathways (Figure 6). These pathways converge at the formation of C3 convertase, which catalyzes downstream events essential for pathogen clearance and immune regulation.

**FIGURE 6:**
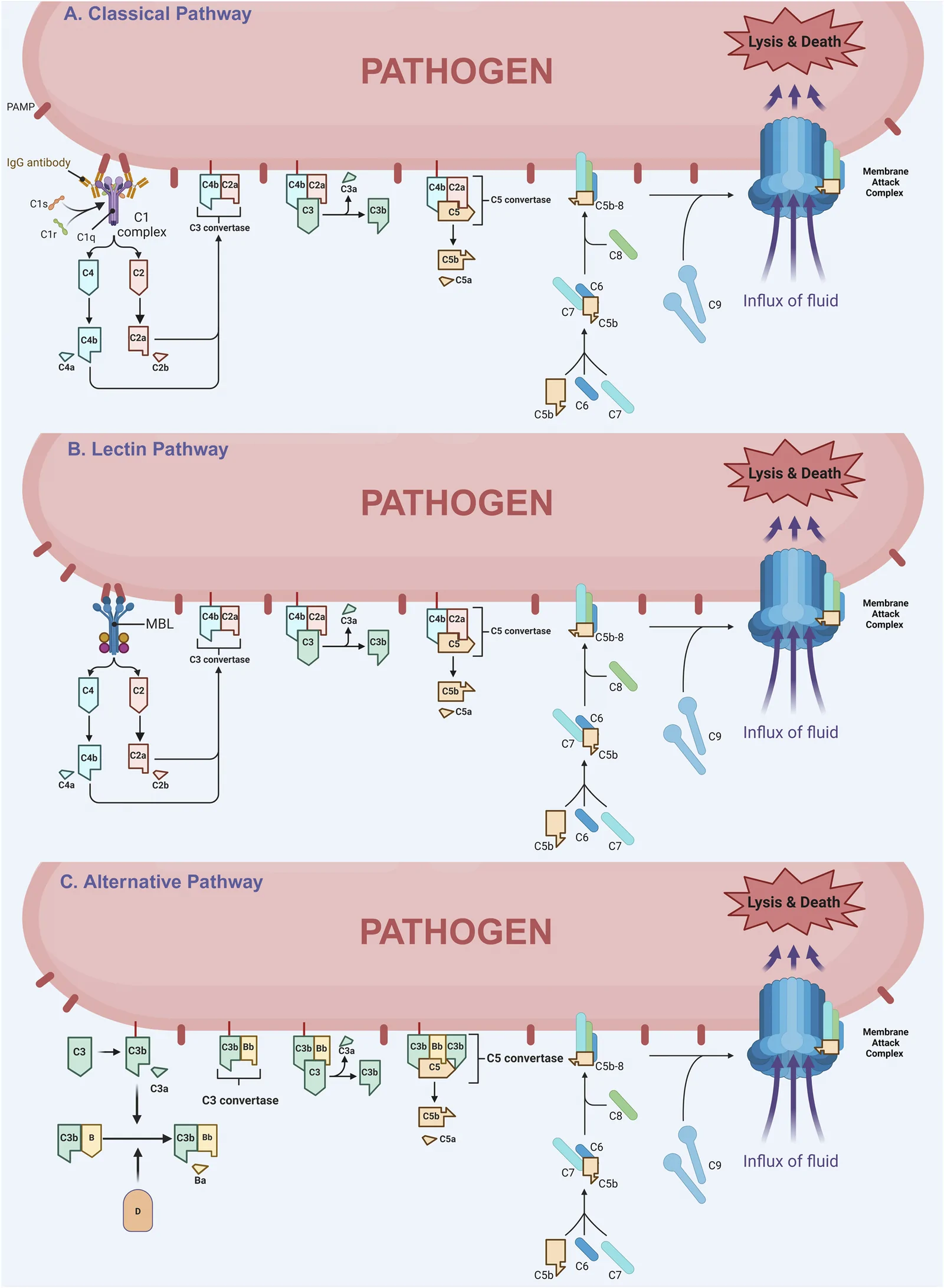
Pathways of complement activation, all leading to the formation of the MAC, resulting in pathogen lysis and death. In the classical pathway **(A)**, PAMPs or IgG antibodies bind to C1q, initiating a cascade involving C1r and C1s, which cleave C4 and C2 to form the C3 convertase (C4bC2a). The lectin pathway **(B)** is triggered by mannose-binding lectin (MBL) binding to carbohydrate moieties on the pathogen surface, activating MASP enzymes that similarly generate C3 convertase (C4bC2a). The alternative pathway **(C)** is initiated spontaneously by hydrolysis of C3, which binds factor B; upon cleavage by factor D, it forms the initial C3 convertase (C3bBb). All three pathways converge at the formation of C5 convertase, which cleaves C5 into C5a and C5b. C5b initiates the assembly of the MAC (C5b-9), composed of C6, C7, C8, and multiple C9 molecules, forming a pore in the pathogen membrane that leads to influx of fluid, osmotic lysis, and cell death. Each pathway highlights distinct upstream triggers but shares a common downstream effector mechanism for microbial elimination. (Created in BioRender. Abdelhamid et al. (2026) https://BioRender.com/j2n5gcv). Abbreviations: MAC, membrane attack complex; PAMP, pathogen-associated molecular pattern; IgG, immunoglobulin G; MBL, mannose-binding lectin; MASP, MBL-associated serine protease.

#### 3.7.1 Complement activation pathways

The complement system is activated through three interconnected pathways: the classical, lectin, and alternative pathways (Figure 6). The classical pathway is initiated by C1q recognition of antigen-antibody complexes (Dunkelberger and Song, 2010), whereas the lectin pathway is triggered by mannose-binding lectin (MBL) and related pattern-recognition molecules binding carbohydrate structures on pathogens or damaged cells (Beltrame et al., 2015). In contrast, the alternative pathway undergoes continuous low-level activation through spontaneous C3 hydrolysis ““tickover”“, providing constant immune surveillance (Pangburn and Müller-Eberhard, 1983). Despite their distinct initiating mechanisms, all three pathways converge at the formation of C3 convertase, leading to generation of C3a and C3b, subsequent C5 cleavage, production of the anaphylatoxins C3a and C5a, and assembly of the membrane attack complex (MAC; C5b-9), which mediates pathogen lysis (Tegla et al., 2011; Morgan, 2016). Beyond host defense, complement activation also regulates inflammation, immune cell recruitment, and tissue remodeling, processes increasingly implicated in hypertension.

#### 3.7.2 The complement system in hypertension

Although traditionally recognized for its role in innate host defense, the complement system is now increasingly appreciated as an active contributor to the pathogenesis of hypertension. Growing evidence from both clinical studies and experimental models demonstrates that complement activation extends beyond antimicrobial immunity to regulate vascular remodeling, endothelial dysfunction, renal injury, and chronic inflammation, all of which are central drivers of elevated blood pressure. Rather than functioning solely as a downstream consequence of inflammation, complement components actively amplify hypertensive pathology through interactions with vascular smooth muscle cells, endothelial cells, macrophages, T cells, and the RAS. Among the complement proteins implicated, C1q contributes to vascular remodeling through activation of Wnt/β-catenin signaling, while C3, the central convergence point of all three complement pathways, has been consistently associated with hypertension onset and progression. Furthermore, the anaphylatoxins C3a and C5a exert complex, context-dependent effects by modulating both innate and adaptive immune responses through their respective receptors, thereby influencing vascular inflammation, tissue injury, and blood pressure regulation. Collectively, these findings position the complement system as a critical mediator linking immune activation to hypertension and highlight complement-targeted therapies as promising strategies for blood pressure control.

##### 3.7.2.1 C1q in hypertension

The Wnt/β-catenin signaling pathway appears to play a rather important role in the pathogenesis of hypertension (Figure 7). Indeed, in VSMCs, binding of C1q to frizzled receptors and their co-receptors, namely, lipoprotein receptor-related protein 5/6 (LRP5/6), activates the canonical Wnt/β-catenin pathway (Naito et al., 2012). This activation involves the stabilization and accumulation of β-catenin in the cytoplasm, facilitated by the inhibition of the destruction complex, which comprises adenomatous polyposis coli, axin, casein kinase 1, and glycogen synthase kinase 3β (Stamos and Weis, 2013). Normally, in the absence of Wnt/β-catenin signaling, β-catenin is phosphorylated by casein kinase 1 and synthase kinase 3β within the destruction complex, leading to its ubiquitination and proteasomal degradation (Stamos and Weis, 2013). However, upon Wnt/β-catenin pathway activation by C1q, the cytoplasmic dishevelled protein is recruited to provide a platform for the destruction complex to phosphorylate LRP5/6, halting β-catenin phosphorylation and degradation (MacDonald and He, 2012; Mukherjee et al., 2018). Consequently, stabilized β-catenin accumulates in the cytoplasm and translocates into the nucleus, where it forms a transcriptional complex with the T-cell factor/lymphoid enhancer factors (MacDonald et al., 2009). This complex modulates the expression of target genes involved in cell proliferation and vascular remodeling, including cyclin D1 and c-myc, promoting VSMC proliferation (Quasnichka et al., 2006).

**FIGURE 7:**
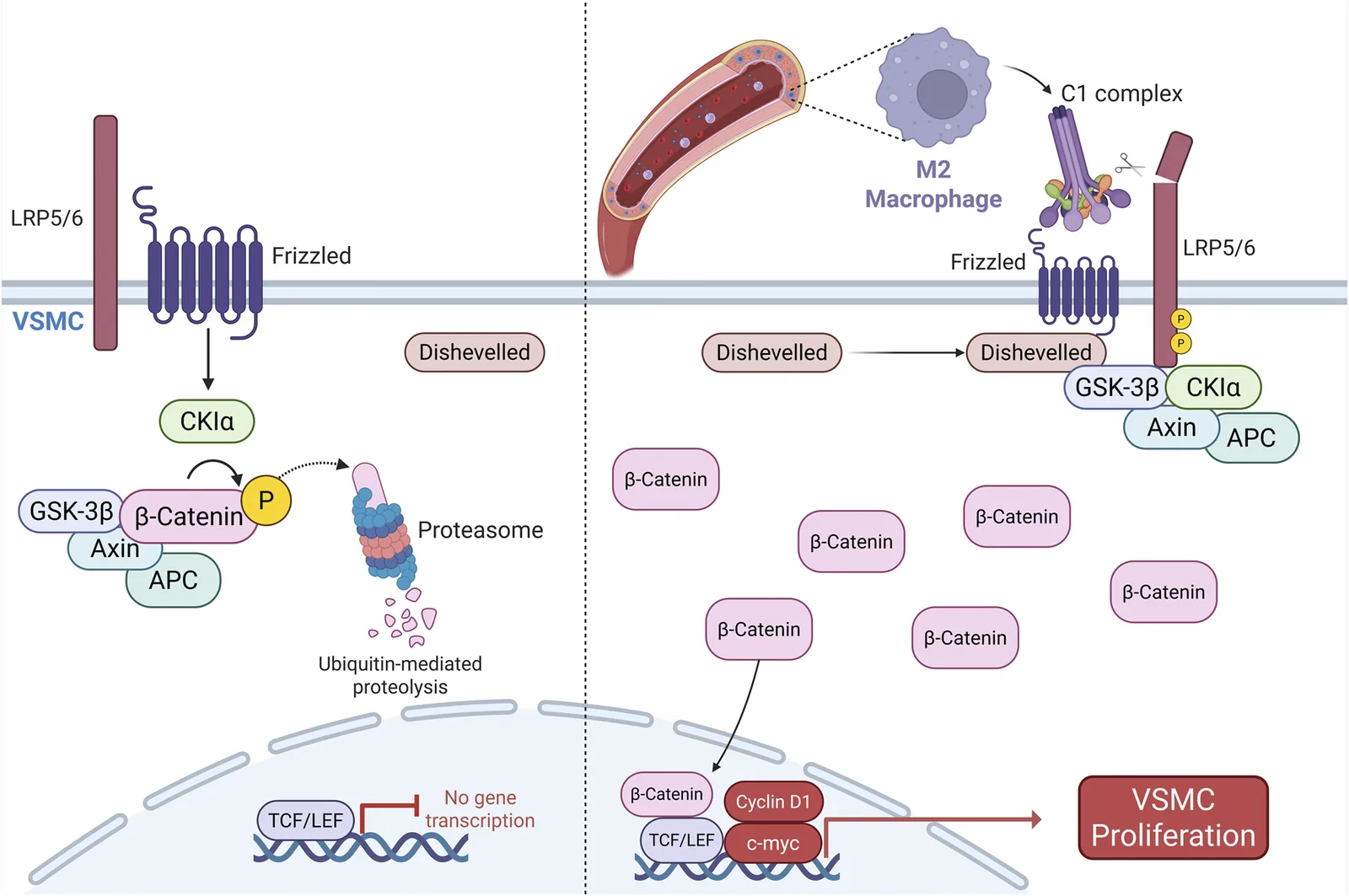
The dual regulation of the Wnt/β-catenin signaling pathway in VSMCs. The figure highlights the contrast between inactive and activated states. In the absence of Wnt signaling (left panel), β-catenin is constitutively phosphorylated by a destruction complex composed of GSK-3β, Axin, APC, and CK1α, leading to its ubiquitin-mediated proteolysis by the proteasome. This prevents β-catenin from translocating to the nucleus, resulting in no transcriptional activation of target genes such as cyclin D1 and c-myc, and thus maintaining VSMC quiescence. Upon Wnt ligand binding to Frizzled and LRP5/6 receptors (right panel), Dishevelled is recruited and inhibits the destruction complex, preventing β-catenin degradation. Stabilized β-catenin accumulates in the cytoplasm and translocates to the nucleus, where it binds TCF/LEF transcription factors to activate expression of pro-proliferative genes including cyclin D1 and c-myc, promoting VSMC proliferation. Additionally, an M2 macrophage-derived C1 complex is shown to enhance this pathway by facilitating Frizzled activation, suggesting a role for immune modulation in Wnt signaling during vascular remodeling. (Created in BioRender. Abdelhamid et al. (2026) https://BioRender.com/hjod2be). Abbreviations: VSMC, vascular smooth muscle cell; GSK-3β, glycogen synthase kinase 3 beta; APC, adenomatous polyposis coli; CK1α, casein kinase 1 alpha; LRP5/6, lipoprotein receptor-related protein 5/6; TCF, T-cell factor; LEF, lymphoid enhancer factor.

M2 macrophages recruited into the aortic adventitia following Ang II-infusion are a source of C1q. This molecule activates the Wnt/β-catenin signaling cascade, driving VSMC proliferation and hypertension (Sumida et al., 2015). This signaling cascade is further amplified by the VSMC-derived C1r/s cleavage of LRP5/6 (Naito et al., 2012). The enhanced proliferation of VSMC contributes to arterial stiffness and vascular remodeling, leading to increased vascular resistance and elevated blood pressure characteristic of hypertension (Brown et al., 2018). As such, activation and amplification of the Wnt/β-catenin pathway by the C1 complex in VSMCs may therefore offer novel therapeutic strategies for managing hypertension and preventing associated cardiovascular complications. Indeed, depletion of M2 macrophages, administration of C1 inhibitors, and genetic ablation of the Wnt/β-catenin signaling elements have shown success in suppressing Ang II-induced activation of this signaling pathway and therefore VSMC proliferation (Allison, 2015; Sumida et al., 2015).

##### 3.7.2.2 C3 in hypertension

The complement protein C3 is integral to all three complement cascades, and contributes to hypertension through several mechanisms. A prospective study following individuals for 15 years revealed elevated levels of both C3 and C4 in those with hypertension at baseline. Surprisingly, for those without hypertension at baseline, the incidence of newfound hypertension and changes in blood pressure over the follow-up period was proportional to the initial levels of C3 but not C4 (Engström et al., 2007). This finding aligns with another study involving over seven thousand individuals, which similarly reported increased C3 plasma concentrations in prehypertensive patients (Bao et al., 2017). These intriguing observations, suggesting the absence of C4’s involvement in the initiation of hypertension, beg the question whether the alternative pathway is responsible for the onset and development of hypertension. The urgency to address this matter grows with the discovery that complement factor H polymorphisms, specific to the alternative pathway, escalate the risk of incident coronary heart disease and ischemic stroke, with the magnitude of association dependent on hypertension status (Volcik et al., 2008).

Increased concentrations of C3 have also been demonstrated to be primary initiators of the renin-angiotensin-aldosterone system (Zhou et al., 2013). This system contributes to hypertension by promoting vasoconstriction, sodium retention, and plasma volume expansion (Manrique et al., 2009). In turn, renin has been shown to cleave C3 into C3b and C3a in a manner identical to C3 convertase, providing a direct mechanistic link between RAS and complement activation (Kristoffersson et al., 2025). Moreover, the increased production of C3 by perivascular adipose tissue in hypertensive DOCA-salt models contributes to vascular remodeling and promotes the polarization towards a pro-inflammatory macrophage phenotype. Taken together, the aforementioned effects of C3 ultimately result in renal and vascular damage, and consequently hypertension (Ruan et al., 2010; Ruan et al., 2015). Therefore, targeting C3 production may offer therapeutic benefits.

##### 3.7.2.3 Anaphylatoxins in hypertension

Several studies highlight the dual effect of anaphylatoxins. They can either mediate or mitigate increased blood pressure and the consequent end-organ damage. These contradicting effects are attributed to divergent outcomes of their receptors’ activation. For instance, depending on where they are expressed, C3aRs can either promote pro-inflammatory or anti-inflammatory effects (Gao et al., 2020). Indeed, in T cells, C3aR activation contributes to an anti-inflammatory effect, important for maintaining T cell survival and homeostasis (Liszewski et al., 2013). Accordingly, C3aR-deficient mice show a tendency towards increased cardiac and renal injury following induction of hypertension via Ang II infusion (Zhang et al., 2014a). This highlights the importance of C3aRs to dampen injury during hypertension.

This interplay of receptors also extends to those of C5a, where C5aR1 elicits pro-inflammatory effects, but the controversial role of C5aR2 exhibits both pro-inflammatory and anti-inflammatory outcomes (Li et al., 2013). In contrast to C3a, the interaction of C5a with its intracellular receptor C5aR1 within T cells prompts a surge in ROS production, subsequently triggering the synthesis and assembly of NLRP3 (Jimenez-Duran and Triantafilou, 2021). This inflammasome steers T cells towards the pro-inflammatory Th1 phenotype, characterized by the release of cytokines such as IFN-γ and TNF-a, ultimately contributing to kidney damage associated with hypertension (Zhang J. et al., 2014; Martynova et al., 2022). Indeed, elevated levels of C5a correlate with heightened blood pressure in human subjects, and Ang II-induced hypertension in mice is associated with an escalation in systemic anaphylatoxin production (Zhang et al., 2014a).

The impact of C5a on hypertension becomes evident through observations in C5aR1-deficient mice, which displayed diminished cardiac remodeling and inflammation following Ang II infusion. Similarly, the administration of an anti-C5a monoclonal antibody or the C5aR1 antagonists yielded outcomes akin to those of C5aR-deficient mice. Not only did C5aR1 antagonists mitigate endothelial dysfunction, but also reduced hypertensive cardiac injury, along with decreased fibrosis and hypertrophy of the ventricles (Iyer et al., 2011; Zhang et al., 2014b). Interestingly, contrary to previous findings, a study reported that although the infusion of Ang II in C5aR1-deficient mice ameliorates hypertensive renal injury, it exacerbates cardiac damage, marked by increased cardiac fibrosis and heart weight (Weiss et al., 2016). The exact explanation for this remains elusive, but it can be postulated to be due to variations in the distribution of C5aR1 and C5aR2 expression across the body tissues. To clarify, it is plausible that some C5aR1-deficient tissues may exhibit heightened anti-inflammatory C5aR2 expression and therefore demonstrate protective effects, in contrast to tissues expressing pro-inflammatory C5aR2 or having low expression of this receptor. For instance, the kidneys might exhibit increased expression of anti-inflammatory C5aR2, while the heart may have a higher expression of pro-inflammatory C5aR1 or C5aR2. However, this remains a proposed explanation, and it is therefore of great importance to understand the precise interplay of the distribution patterns of C5aR1 and C5aR2 and the possible consequences of targeting them.

The roles of C3a and C5a have also been implicated in hypertension through their effects on regulatory T (T_reg_) cells. These specialized immune cells, characterized by their expression of CD4 and CD25, are crucial for immune regulation, suppressing exaggerated immune responses and preserving immune tolerance (Vignali et al., 2008). The immunosuppressive role of such cells is attributed to their release of anti-inflammatory cytokines such as interluekin-10, interleukin-35, and TGF-β1 (Vignali et al., 2008; Kassan et al., 2013). The development of T_reg_ cells in the thymus hinges on the cytokine interleukin-2 (IL-2), which sustains the high expression of their distinctive transcription factor Foxp3 (Rudensky, 2011). Additionally, IL-2 plays a crucial role in regulating the proliferation and maintenance of T_reg_ cells in peripheral tissues (Ross and Cantrell, 2018).

Multiple studies have elucidated the involvement of T_reg_ cells in alleviating cardiac and renal injury (Kvakan et al., 2009; Barhoumi et al., 2011; Matrougui et al., 2011; Kasal et al., 2012). Certainly, administering IL-2/anti-IL-2 immune complex to hypertensive mice has been found to boost T_reg_ cells and ameliorate aortic stiffening (Majeed et al., 2014). However, their dysregulation in hypertension appears to be influenced by the actions of the anaphylatoxins C3a and C5a; their interaction with their receptors C3aR and C5aR1, respectively, inhibits the function of circulating T_reg_ cells (Kwan et al., 2013; Strainic et al., 2013). Indeed, the expression of C3aR and C5aR1 on T_reg_ cells has been observed to rise in Ang II-induced hypertension (Chen et al., 2018). Remarkably, C3aR and C5aR1 double knockout mice exhibited decreased blood pressure in response to Ang II by restoring Foxp3 expression in activated T cells. Furthermore, the subsequent adoptive transfer of T_reg_ cells deficient in C3aR and C5aR1 revealed protective effects against blood pressure elevation and renal injury, and these effects were nullified upon T_reg_ depletion using CD25 neutralizing antibodies (Chen et al., 2018).

Although the aforementioned findings confirm the involvement of C3a and C5a in hypertension by suppressing T_reg_, delving deeper into this interaction is essential to elucidate its exact mechanism. This necessity stems from that while hypertension typically correlates with a diminished T_reg_ population compared to normotensive counterparts (Chen et al., 2018), a discrepancy arises. Contrary to expectations, a study on hypertensive mouse models exhibited an upsurge in T_reg_ within the kidneys, suggesting a potential protective response to elevated blood pressure (Krebs et al., 2014). Further investigation is essential to reconcile these contrasting findings and ensure the effectiveness of targeting the interaction between anaphylatoxins and T_reg_ cells in the treatment of hypertension.

### 3.8 Sex differences in innate immune mechanisms of hypertension

Sex and sex hormones are known to affect a multitude of aspects pertraining to cardiovascular physiology and pathophysiology, including vascular function, cardiovascular disease susceptibility, and responses to treatment (Eid et al., 2007; Huxley, 2007; Boese et al., 2017; Dehaini et al., 2018; Fardoun M. et al., 2020; Wehbe et al., 2020; Ueda et al., 2021; Drury et al., 2024; Stamellou et al., 2024). For instance, we have previously demonstrated that the female hormone, estrogen, modulates vascular adrenoceptor expression and function in human arteriolar smooth muscle cells (Eid et al., 2007; Fardoun M. M. et al., 2020). Indeed, our results showed how sex contributes to the pathophysiology of Raynaud’s phenomenon. We also recently discussed how sex may bias responsiveness to antihpertensive agents (Yaacoub et al., 2026). Within this broad context, it is important to note that sex differences in hypertension and immune regulation have also increasingly attracted attention as an important biological dimension of cardiovascular disease and, therefore, warrant consideration when examining the contribution of innate immunity to blood-pressure regulation. Men generally develop hypertension earlier in life than premenopausal women, whereas this sex difference becomes less pronounced or may reverse after menopause, suggesting important contributions from sex hormones, sex chromosomes, and tissue-specific immune regulation (Sandberg et al., 2015; Ramirez and Sullivan, 2018; Drury et al., 2024). At the molecular level, TLR signaling is sexually dimorphic, with differences in TLR expression and responsiveness potentially contributing to sex-specific inflammatory and blood-pressure responses (Sandberg et al., 2015; Dela Justina et al., 2020). Experimental and clinical literature indicates that TLR activation may contribute differently to blood-pressure regulation in males and females, although the precise mechanisms and relevance to individual TLRs remain incompletely defined (Dela Justina et al., 2020). Macrophage responses are similarly sex-dependent: experimental hypertension and metabolic stress can produce different macrophage phenotypes and inflammatory transcriptional programs in males and females, with sex-specific differences in macrophage accumulation and phenotype detectable even before the development of overt hypertension (Kumar et al., 2021). NK-cell biology is also strongly influenced by sex, although its specific contribution to sex differences in hypertension remains less well characterized than that of TLRs and macrophages (Sandberg et al., 2015). Sex hormones and sex chromosomes can alter innate immune-cell activation, cytokine production, and inflammatory signaling, providing plausible mechanisms through which the same hypertensive stimulus may produce different immune responses between males and females (Sandberg et al., 2015; Ramirez and Sullivan, 2018).

Given these biological differences, an important question is whether interventions targeting innate immune pathways produce different effects on blood-pressure control in males and females. Future studies should therefore incorporate sex as an important biological variable when evaluating immune-targeted therapies and should assess sex-specific differences in innate immune-cell abundance, activation state, tissue recruitment, receptor signaling, and therapeutic response. Such approaches may help determine whether immunomodulatory interventions have differential efficacy or safety profiles between men and women and may ultimately contribute to the identification of sex-specific hypertension endotypes and more individualized treatment strategies.

#### 3.8.1 Relative strength of evidence across innate immune pathways

The available evidence does not support treating all innate immune pathways as equally established contributors to systemic hypertension. Among the pathways reviewed, TLR4, NLRP3 inflammasome signaling, and dendritic-cell-mediated antigen presentation currently have the strongest and most consistent causal evidence, although important uncertainties remain. TLR4 has been implicated across multiple experimental models, with genetic deletion or pharmacological inhibition attenuating hypertension or vascular dysfunction in several settings, and human genetic and observational studies providing additional, albeit less definitive, support. NLRP3 similarly has substantial mechanistic support: its components are activated in experimental hypertension, while genetic deletion or pharmacological inhibition can reduce blood pressure and/or renal and vascular injury in several models, although effects are not uniformly reproduced across all hypertensive stimuli. Dendritic cells also have relatively strong causal evidence, as disruption of B7/CD28 costimulation, manipulation of dendritic-cell function, and adoptive-transfer experiments have demonstrated that these cells can contribute directly to experimental hypertension; importantly, altered dendritic-cell populations and activation states have also been documented in humans with hypertension. In comparison, monocytes/macrophages and neutrophils have substantial experimental and observational support but encompass highly heterogeneous populations and therefore present greater challenges in identifying specific pathogenic subsets or targets. The evidence for complement and NK cells is currently less mature in relation to systemic hypertension, with promising mechanistic and experimental observations but fewer studies demonstrating consistent blood-pressure causality across models and limited therapeutic validation. Thus, the field is better represented as a hierarchy of evidentiary maturity rather than a collection of equivalent pathways: TLR4, NLRP3, and dendritic-cell pathways have relatively strong causal support; monocyte/macrophage and neutrophil pathways have substantial but heterogeneous evidence; and NK-cell and complement pathways remain comparatively exploratory. This distinction is important for prioritizing future research and avoiding premature translation of promising biological associations into therapeutic claims.

#### 3.8.2 Translational challenges and clinical perspectives

Although accumulating preclinical evidence supports targeting innate immune pathways as a therapeutic strategy for hypertension, translation into clinical blood pressure reduction has thus far been limited. One of the most notable examples is the Canakinumab Anti-inflammatory Thrombosis Outcomes Study (CANTOS), which evaluated canakinumab, a monoclonal antibody targeting IL-1β, in patients with previous myocardial infarction and elevated high-sensitivity C-reactive protein (Ridker et al., 2017). While IL-1β inhibition significantly reduced systemic inflammation and lowered the incidence of recurrent cardiovascular events, it did not produce clinically meaningful reductions in blood pressure. Similarly, a dedicated secondary analysis of the CANTOS cohort demonstrated that canakinumab had no significant effect on systolic or diastolic blood pressure despite effectively suppressing inflammatory biomarkers (Rothman et al., 2020). These studies suggest that inhibition of a single inflammatory pathway may be insufficient to reverse the complex mechanisms underlying established hypertension.

Several factors may explain the discrepancy between encouraging preclinical findings and the limited success of anti-inflammatory therapies in human hypertension. Experimental models, particularly Ang II-induced hypertension, often represent highly inflammatory disease states that may not fully capture the heterogeneous pathophysiology observed in patients. In contrast, human hypertension arises from complex interactions among genetic susceptibility, neurohormonal activation, renal dysfunction, metabolic abnormalities, environmental factors, and immune responses (Murray et al., 2021). Consequently, innate immune activation may represent only one component of disease progression, and considerable redundancy among inflammatory pathways may limit the effectiveness of targeting individual cytokines or receptors. Accordingly, anti-inflammatory therapies may provide greater benefit in carefully selected patient populations characterized by heightened inflammatory activity rather than in unselected hypertensive cohorts.

These findings underscore the need for precision medicine approaches that incorporate immune phenotyping and biomarker-guided patient selection to identify individuals most likely to benefit from immunomodulatory therapies. It is worth mentioning that interindividual variability in antihypertensive drug efficacy and tolerability is influenced not only by comorbidities but also by biological sex and genetic background. Indeed, we recently discussed the sex-biased disparities in the prevalence, pathophysiology as well as pharmacotherapeutic outcomes in the context of hypertension (Yaacoub et al., 2026). Notably, despite its well-established vasodilatory and vasoprotective effects, the sex hormone, estrogen, has also been proposed to elicit a vasoconstrictive effect (Fardoun M. et al., 2020; Drury et al., 2024; Riedel et al., 2026), although some studies differentiate between responses in veins or arteries (Haas et al., 2007). We have also previously discussed how genetic and epigenetic factors may likewise be implicated in interindividual difference in hypertension suscpeibility or responses to anti-hypertensive therapies (Yaacoub et al., 2024; El Cheikh et al., 2025). Although the effects of these factors are often modest and the clinical evidence is heterogenous, thereby limiting their imeidate clinical applicability, it is nonetheless important to acknowledge the importance of incorporating sex-aware and pharmacogenomic approaches into the personalization of antihypetensive therapy. Moreover, advances in artificial intelligence and machine learning are expected to accelerate these paradigms by integrating complex clinical and multi-omics datasets to identify hypertension endotypes, predict therapeutic responsiveness, and facilitate the discovery and development of novel immunomodulatory therapies targeting innate immune pathways (Abdelhamid et al., 2026). Future clinical trials should therefore focus not only on evaluating novel innate immune targets but also on identifying hypertension endotypes in which immune dysregulation represents a dominant pathogenic mechanism.

## 4 Conclusion

This review demonstrates that the innate immune system plays a significant contributing role in the development of hypertension, rather than a mere consequence. Indeed, this recognition transcends the previous paradigm of focusing solely on hemodynamic control. The interplay between PRRs, particularly TLRs and NLRs, and the coordinated action of immune cells (monocytes/macrophages, DCs, and neutrophils) construct a powerful pro-inflammatory landscape within the vasculature (Figure 8). This perspective necessitates a shift in therapeutic strategies. By targeting the core mechanisms driving innate immune dysregulation in hypertension, novel interventions can be designed to achieve targeted and precise immune modulation, offering the potential for long-term cardiovascular protection.

**FIGURE 8:**
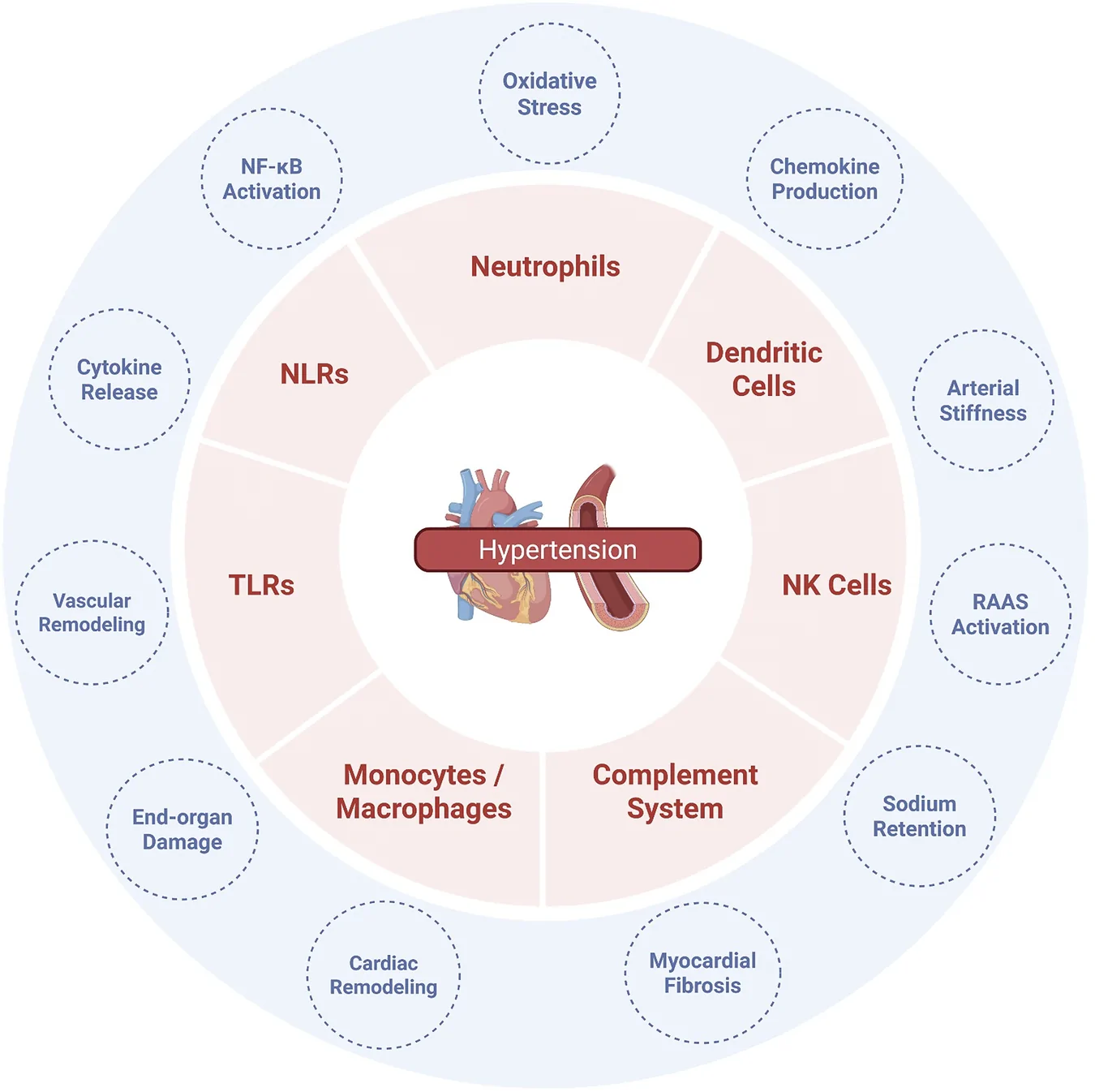
Summary of the contribution of innate immune components to the development and progression of hypertension. The schematic summarizes the contribution of the major innate immune system components to the pathogenesis of hypertension. The inner circle depicts the principal innate immune pathways involved in hypertension, including TLRs, NLRs, monocytes/macrophages, DCs, neutrophils, NK cells, and the complement system. These components interact through interconnected inflammatory signaling networks, promoting oxidative stress, chemokine production, arterial stiffness, RAAS activation, sodium retention, myocardial fibrosis, cardiac remodeling end-organ damage vascular remodeling, cytokine release, and NF-κB activation. Abbreviations: TLR, toll-like receptor; NLR, nucleotide-binding oligomerization domain-like receptor; NK, natural killer; NF-κB, nuclear factor kappa B; NO, nitric oxide. (Created in BioRender. Abdelhamid et al. (2026) https://BioRender.com/i3h6u66).

While preclinical studies robustly support the targeting of innate immune components in hypertension, translation to human subjects remains a critical frontier. Indeed, much of the mechanistic evidence has been derived from Ang II-induced experimental models, and additional human studies are needed to clarify whether innate immune activation is a primary driver, an amplifier, or a consequence of hypertensive disease. Advanced diagnostics capable of stratifying hypertensive patients based on their innate immune profiles would further refine personalized treatment approaches. The therapeutic implications of these findings should nevertheless be interpreted according to the markedly different levels of clinical maturity across innate immune targets. At present, direct inhibition of TLRs, NLRP3, or specific innate immune-cell populations remains largely investigational for systemic hypertension, with most evidence derived from genetic or pharmacological experiments in animal models. NLRP3 is arguably among the more advanced innate immune targets, with selective inhibitors such as dapansutrile (OLT1177) reaching clinical development in inflammatory and cardiometabolic diseases. However, clinical evidence has not yet demonstrated a clear antihypertensive effect. Similarly, although IL-1β inhibition has shown cardiovascular benefits, the CANTOS trial found no meaningful reduction in blood pressure, highlighting that reducing inflammation does not necessarily translate into antihypertensive efficacy. In contrast, TLR4 inhibition and therapies targeting dendritic-cell, macrophage, or NK-cell function remain largely preclinical. Among these, dendritic-cell pathways may offer particular translational potential, given evidence from experimental models and emerging human data linking altered dendritic-cell activity with hypertension. Adoptive cellular therapies aimed at modifying macrophage or dendritic-cell behavior should therefore currently be regarded as speculative rather than near-term therapeutic options, given the complexity, heterogeneity, and potential systemic consequences of manipulating innate immune cells.

Overall, the most realistic near-term strategy is unlikely to be broad immunosuppression; rather, it will involve identifying specific inflammatory endotypes and selectively targeting pathways with demonstrated causal relevance, while preserving host defense and minimizing infection or immune-related toxicity. Future hypertension trials should therefore incorporate blood-pressure endpoints alongside immune biomarkers and target-organ outcomes to determine whether modulation of innate immunity provides genuine antihypertensive benefit rather than merely reducing inflammation.

## Glossary

Ang II: Angiotensin II
APCs: antigen-presenting cells
C3aR: C3a receptor
C5aR1: C5a receptor 1
C5aR2: C5a receptor 2
CCR2: CC chemokine receptor 2
CVD: cardiovascular disease
DAMPs: damage-associated molecular patterns
DCs: dendritic cells
DNases: deoxyribonucleases
HO-1: heme oxygenase-1
IFN-γ: interferon gamma
IL-1β: interleukin-1 beta
IL-18: interleukin-18
IL-2: interleukin-2
IL-23: interleukin-23
IL-6: interleukin-6
iNOS: inducible nitric oxide synthase
IsoLG: isolevuglandin
KIRs: killer cell immunoglobulin-like receptors
LRP5/6: lipoprotein receptor-related protein 5/6
MASPs: MBL-associated serine proteases
MBL: mannose-binding lectin
MPO: myeloperoxidase
MyD88: myeloid differentiation primary response 88
NLRs: nucleotide-binding oligomerization domain-like receptors
NETs: neutrophil extracellular traps
NF-κB: nuclear factor kappa B
NK: natural killer
NLRP3: NOD-, LRR-, and pyrin domain-containing protein
NO: nitric oxide
PAMPs: pathogen-associated molecular patterns
PAD4: peptidyl arginine deiminase 4
PRRs: pattern recognition receptors
PVN: paraventricular nucleus
ROS: reactive oxygen species
TGF-β: transforming growth factor beta
TIR: toll/interleukin-1 receptor
TLRs: toll-like receptors
TNF-α: tumor necrosis factor-alpha
TRIF: TIR-domain-containing adapter inducing interferon-β
Treg: regulatory T
VCAM-1: vascular cell adhesion molecule-1
VSMCs: vascular smooth muscle cells
